# Active Microphase Separation in Mixtures of Microtubules and Tip-Accumulating Molecular Motors

**DOI:** 10.1103/physrevx.12.031006

**Published:** 2022-07-11

**Authors:** Bezia Lemma, Noah P. Mitchell, Radhika Subramanian, Daniel J. Needleman, Zvonimir Dogic

**Affiliations:** 1 Physics Department, Harvard University, Cambridge, Massachusetts 02138, USA; 2 Physics Department, Brandeis University, Waltham, Massachusetts 02453, USA; 3 Physics Department, University of California, Santa Barbara, California 93106, USA; 4 Kavli Institute for Theoretical Physics, University of California, Santa Barbara, California 93106, USA; 5 Molecular Biology Department, Massachusetts General Hospital Boston, Massachusetts 02114, USA; 6 Genetics Department, Harvard Medical School, Boston, Massachusetts 02115, USA; 7 John A. Paulson School of Engineering and Applied Sciences, Harvard University, Cambridge, Massachusetts 02138, USA; 8 Molecular and Cellular Biology Department, Harvard University, Cambridge, Massachusetts 02138, USA; 9 Center for Computational Biology, Flatiron Institute, New York, New York 10010, USA; 10 Biomolecular Science and Engineering Department, University of California, Santa Barbara, California 93106, USA

**Keywords:** Biological Physics, Fluid Dynamics, Soft Matter

## Abstract

Mixtures of filaments and molecular motors form active materials with diverse dynamical behaviors that vary based on their constituents’ molecular properties. To develop a multiscale of these materials, we map the nonequilibrium phase diagram of microtubules and tip-accumulating kinesin-4 molecular motors. We find that kinesin-4 can drive either global contractions or turbulentlike extensile dynamics, depending on the concentrations of both microtubules and a bundling agent. We also observe a range of spatially heterogeneous nonequilibrium phases, including finite-sized radial asters, 1D wormlike chains, extended 2D bilayers, and system-spanning 3D active foams. Finally, we describe intricate kinetic pathways that yield microphase separated structures and arise from the inherent frustration between the orientational order of filamentous microtubules and the positional order of tip-accumulating molecular motors. Our work reveals a range of novel active states. It also shows that the form of active stresses is not solely dictated by the properties of individual motors and filaments, but is also contingent on the constituent concentrations and spatial arrangement of motors on the filaments.

## I. INTRODUCTION

Active matter, the class of materials composed of motile energy-consuming units, exhibits various nonequilibrium dynamical phases [1–6]. For instance, active Brownian particles form dense clusters that share intriguing similarities with conventional gas-liquid phase coexistence, despite purely repulsive interactions [7–10]. Active matter also exhibits distinct dynamical phases with no equilibrium analogs, such as percolating networks that undergo global contractions and turbulentlike flows observed in extensile cytoskeletal filaments or microscopic swimmers [11–16]. Theoretical tools that predict such macroscopic dynamics from microscopic details are still under development [17–21]. Consequently, there is a lack of knowledge about the landscape of the possible dynamic phases that can arise in active matter systems, and our ability to engineer large-scale dynamics by controlling the behavior of microscopic constituents is in its infancy [22]. One way to address this critical knowledge gap is through experiments that measure detailed nonequilibrium phase diagrams of systems with varied microscopic dynamics.

Motivated by these considerations, we study the self-organization of microtubule filaments driven by tip-accumulating kinesin-4 molecular motors. We measure a nonequilibrium phase diagram, finding not only previously described contracting gels and extensile fluids, but also a range of novel structures, which include localized 1D micellelike asters, extended 2D flat bilayers, monolayer covered condensates, and 3D bilayer-based foamlike networks. These structures are different from previously studied forms of active matter due to the importance of both positional and orientational order. They are reminiscent of the diverse microphase-separated equilibrium phases that self-assemble from chemically heterogeneous amphiphilic molecules [23,24]. However, unlike equilibrium amphiphilic self-assembly, which is driven by the chemical immiscibility of different segments [25], the formation and continuous rearrangement of kinesin-4 and microtubule structures are driven by energy-consuming molecular motors. We collectively name these phenomena *active microphase separation*.

The dimeric kinesin-4 molecular motors used in this study consume energy from ATP hydrolysis to step toward microtubule plus ends, where they accumulate [26–28]. Kinesin-4 is associated with the regulation of the central spindle length during cytokinesis and has been observed at the center of microtubule bilayers formed from *Xenopus* egg extracts [29,30]. Kinesin localization results in the formation of segmented microtubules consisting of a motor-rich segment at the plus end and an adjoining motor-poor segment. Thus, the unique properties of kinesin-4 motors yield a reconfigurable building block in which the motor’s microscopic dynamics encodes the filament spatial heterogeneity, unlike the permanently encoded chemical structure of conventional amphiphiles. Microscopic parameters such as the microtubule length and the kinesin-4 concentration determine the size of the motor-rich domain [28,31]. The plus-end segment can slide along other microtubules to their plus ends, but the mechanism of this motion is not well understood [28,31].

## II. RESULTS

### A. Active asters: Asters self-organize and reconfigure

**FIG. 1.**
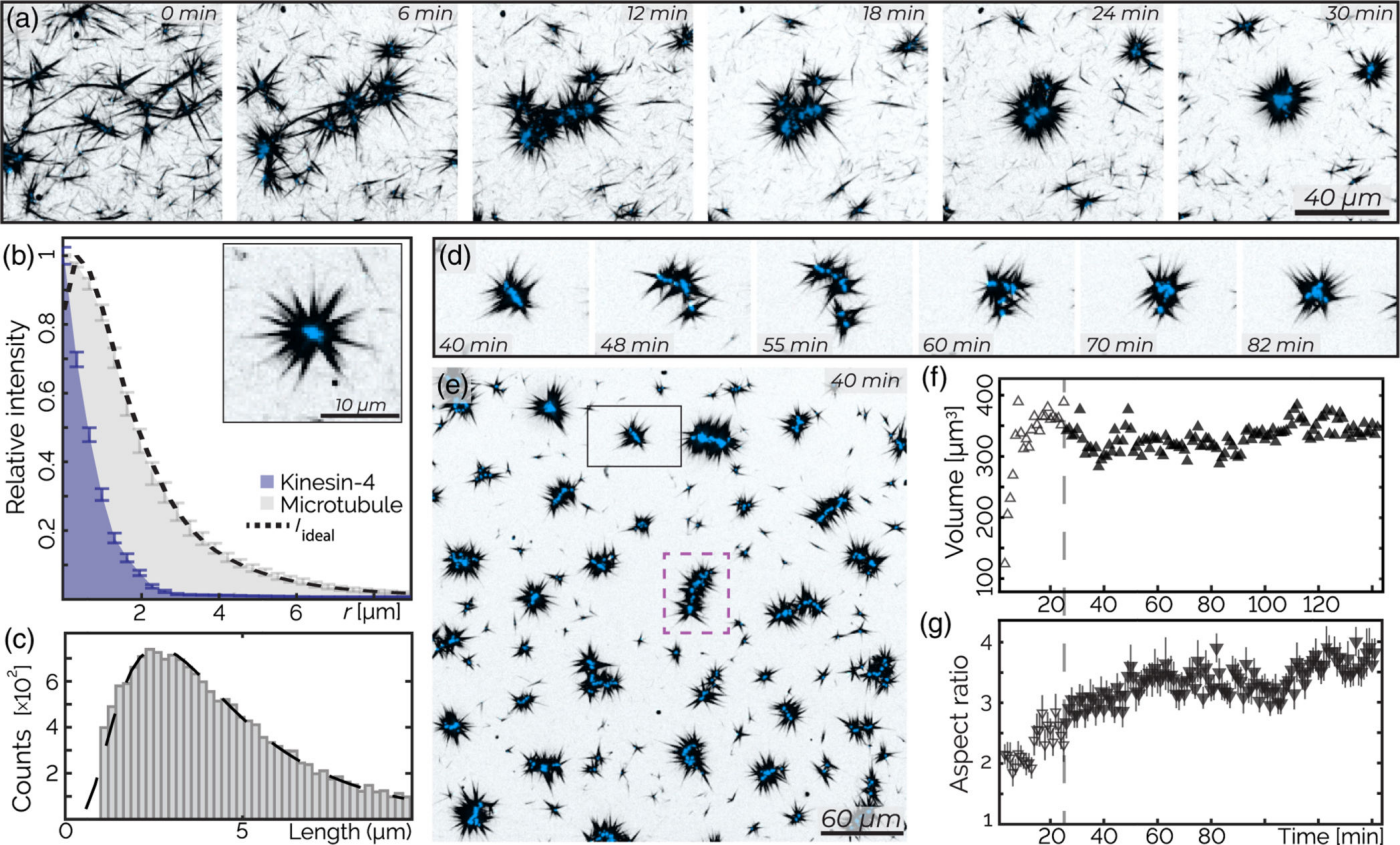
Active asters: Asters self-organize and reconfigure. (a) Kinesin-4 induces rapid assembly of asters. (b) The density profile of microtubules (gray) radially averaged from the z projection of an aster. Predicted structures *I*_ideal_ (dotted black line) based on end-bound kinesin-4 motors, given the measured density profile of kinesin-4 (blue). Bars are the standard error averaged over three similar radial asters. Inset: aster with approximate radial symmetry. (c) Microtubule polydispersity (gray bars) is described by a log-normal distribution (dashed black line, *M* = 1.4, *S* = 0.6, mean 4.9 *μ*m, mode 2.8 *μ*m). (d) Temporal rearrangement of an aster. (e) A large field of view shows fully formed asters. The dashed purple line highlights a wormlike structure. (f) The mean aster volume as a function of time. Open shapes indicate the aster formation regime. (g) The mean major/minor moment ratio of asters over time. Bars represent standard deviation. All images are *z* projections over 6.5 *μ*m; the sample contains 200 nM kinesin-4 (blue) and 400 nM tubulin (black).

We first study the organization of a low concentration of stabilized microtubules by kinesin-4 motors in a thin parallelepiped chamber (see the Appendix). Immediately after mixing, we observe microtubules joined by their ends [Fig. 1(a), 0 min]. Within the first approximately 10 min, collections of microtubules continue to merge with each other, while labeled kinesin-4 clusters become visible at locations where filaments join [Fig. 1(a), 6–12 min]. Subsequently, the nascent kinesin clusters merge with each other, forming increasingly better-defined radial structures [Fig. 1(a), 18–24 min]. The intensity of the motor-rich clusters located at the aster core increases, indicating a continual accumulation of motors. Within 30 min, the majority of microtubules condense into radial star-shaped asters with well-defined kinesin-4 cores at their centers [Fig. 1(a), 30 min].

To understand the aster structure, we measure the density profile of radially symmetric asters from 3D confocal images [Fig. 1(b)]. The kinesin core has a radius of approximately 1 *μ*m, while the microtubule profile spans approximately 10 *μ*m radially outward. We hypothesize that microtubules are anchored to the aster core by their tips. To test this proposition, we model the aster structure by convolving the measured microtubule length distribution [Fig. 1(c)] with the intensity profile of the kinesin core (Supplemental Material [32]). This convolution yields a radially averaged microtubule profile that closely matches the experiments [Fig. 1(b), dashed line], which is consistent with our hypothesis.

After their formation, asters continue to evolve by merging with each other and undergoing internal rearrangements [Fig. 1(d)]. Over time, this yields elongated wormlike structures [Fig. 1(e), Video S1]. To characterize such dynamics, we measure the mean three-dimensional moments of the kinesin-rich aster cores. The average ratio between the major and minor moments increases twofold, while the mean volume of asters remains approximately constant [Figs. 1(f) and 1(g)].

### B. Contracting gel: Globally contracting networks generate bilayer structures

**FIG. 2.**
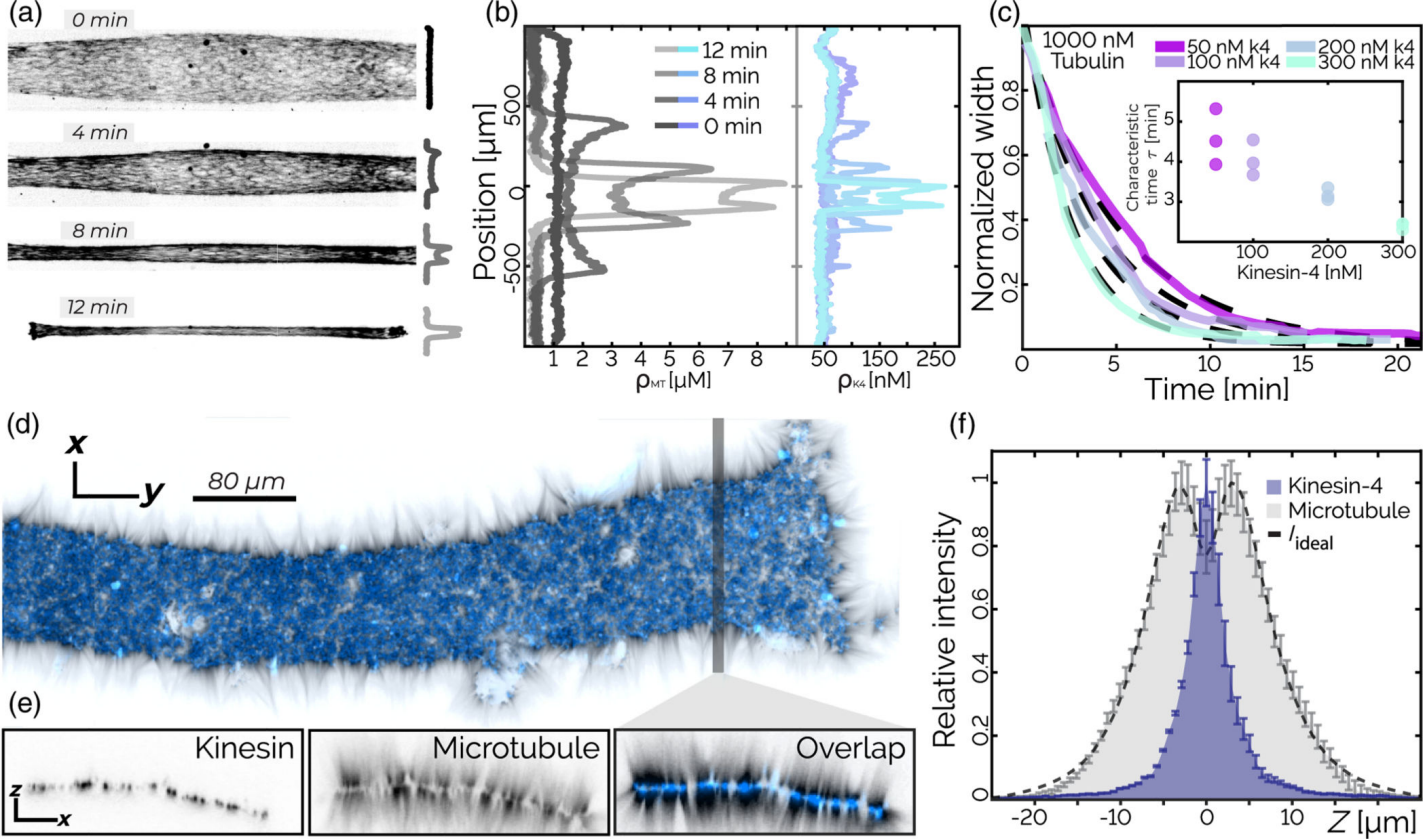
Contracting gel: Globally contracting networks generate bilayer structures. (a) Kinesin-4-driven global contraction of labeled microtubules. (b) Microtubule and kinesin concentration as a function of the position along the chamber short axis reveals nonuniform density growth, with peaks at the sample edges. (c) The normalized width *W*_*n*_(*t*) of a contracting network decays over time. Dashed lines are fits of Eq. (1). Inset: Contraction timescale *τ* decreases with kinesin concentration. Error bars indicate the standard error (*n* = 3). (d) The final structure of the contracted bilayer consists of a kinesin 2D sheet (blue) with microtubules (black) anchored to the surface and pointing along its normal. (e) *x-z* resliced at the shaded line. (f) Fluorescence intensity profile along the surface normal. The predicted microtubule fluorescence *I*_ideal_ (dotted black line) agrees with the measured fluorescence. Bars indicate the standard error over 20 sections of 3 *μ*m width.

By increasing tubulin concentration above 1 *μ*M, we observe the emergence of new dynamics. Instead of forming locally condensed asters, the system globally contracts into a single structure [Fig. 2(a), Video S2]. Material density is highest at the boundaries of the contracting network [Fig. 2(b)], similar to dynein-induced contractions studied in cell extracts and purified systems [16,38]. We track the contracting network width *W*(*t*) over time *t*. The normalized width $W_{n}(t)=W(t)/W(0)$ is described by an exponential function:

(1) $$W_{n}(t)\approx W_{n}^{\infty }+e^{\left[-\left(t-t_{0}\right)\right]/\tau }\left(1-W_{n}^{\infty }\right),$$

where *t*_0_ is a time offset, $W_{n}^{\infty }$ is the final normalized width, and *τ* is the contraction timescale [Fig. 2(c)]. *τ* increases with increasing kinesin concentration [Fig. 2(c)] and decreases with increasing initial tubulin concentration (Fig. S1).

Examination of the final contracted state reveals a well-defined bilayer structure in which the kinesin motors form an extended 2D sheet, with microtubules protruding from both sides of the sheet, pointing along the surface normal [Figs. 2(d) and 2(e)]. In analogy to asters, we hypothesize that microtubules are anchored to the 2D kinesin sheet by their tips. We model the bilayer structure by convolving the measured length distribution of microtubules with the kinesin intensity profile along the surface normal (Supplemental Material [32]). The model of the bilayer structure closely matches the experimentally measured density profile [Fig. 2(f)]. Thus, our analysis suggests that microtubules are connected to the high-density kinesin layer by their plus ends, with their minus ends pointing outward. How an initially disordered contracting network transforms into a late-stage bilayer structure remains to be studied.

**FIG. 3.**
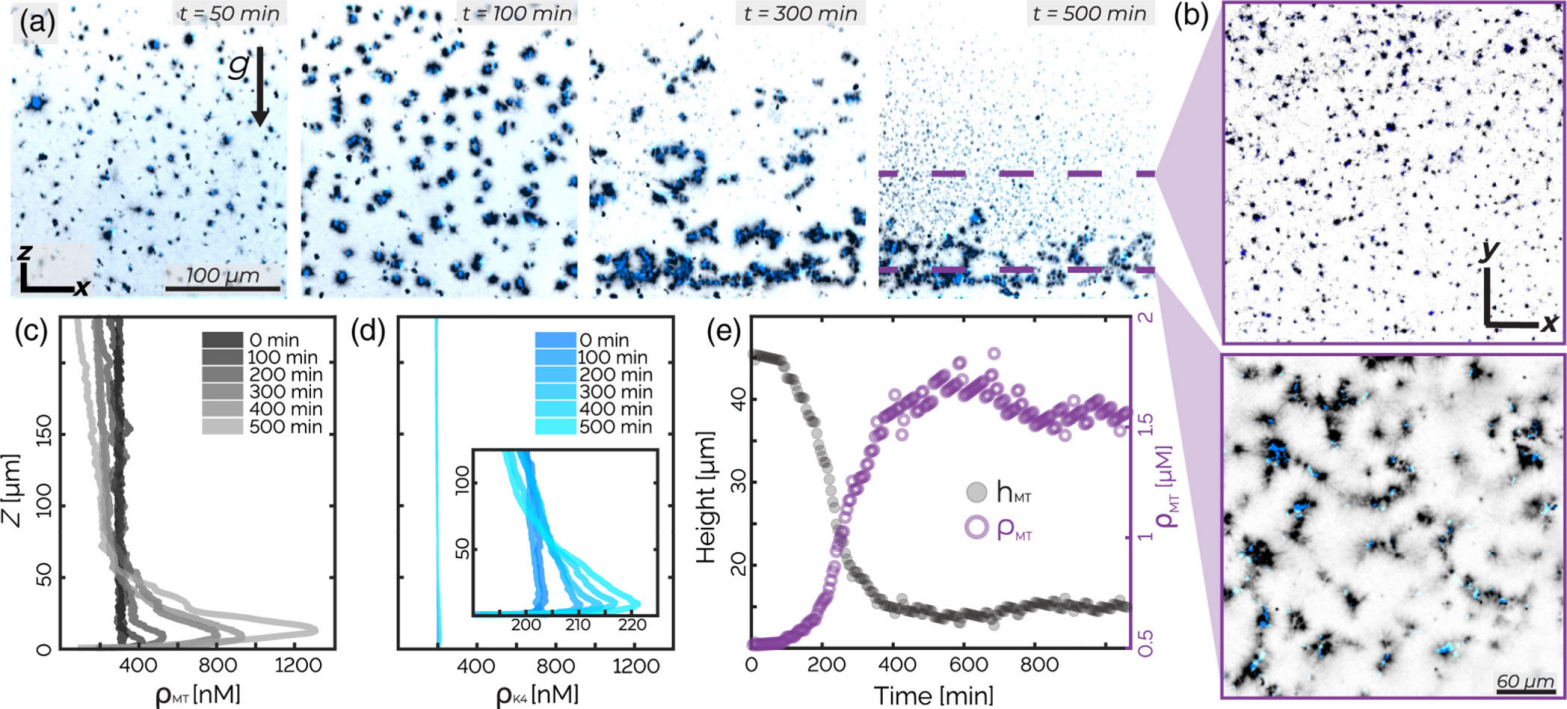
Active asters: Initial conditions determine steady-state dynamics. (a) *x-z* plane images show the aster assembly and sedimentation. The arrow indicates gravity; *x-y* is the imaging plane. (b) Aster images in the *x-y* at two different heights at 500 min. (c),(d) Temporal evolution of the density *z* profiles of microtubules *ρ*_MT_ and kinesin *ρ*_K4_ illustrate material sedimentation. (e) The average microtubule density (purple open circles) below the sedimentation height (black circles) as a function of time. The effective microtubule concentration is higher than what is used in Fig. 2, yet no global contraction occurs.

We show that increasing the microtubule concentration induces a transition from local asters to large-scale bilayers. To investigate the importance of initial conditions, we test if increasing the concentration of fully formed asters leads to a similar transition. We prepare a sample with a low filament concentration in a tall sample chamber (250 *μ*m), which leads to the formation of asters throughout the volume. Once formed, large asters sediment into a dense, approximately 50-*μ*m-thick layer, which has an average tubulin density above 1 *μ*M [Figs. 3(b)–3(d)]. Uniformly dispersed samples prepared at such concentrations contract into bilayers. However, the sedimented asters do not contract into a single structure. Instead, they form a dense, continuously rearranging network [Fig. 3(e), Video S3]. The lack of global contraction demonstrates that the form of the long-term steady-state structures depends not only on the constituents local concentration, but also on the sample history. Intriguingly, in contrast to microtubules, a significant fraction of the kinesin does not incorporate into the asters.

### C. Contracting gel: Contractions yield nematic alignment and surface roughening

**FIG. 4.**
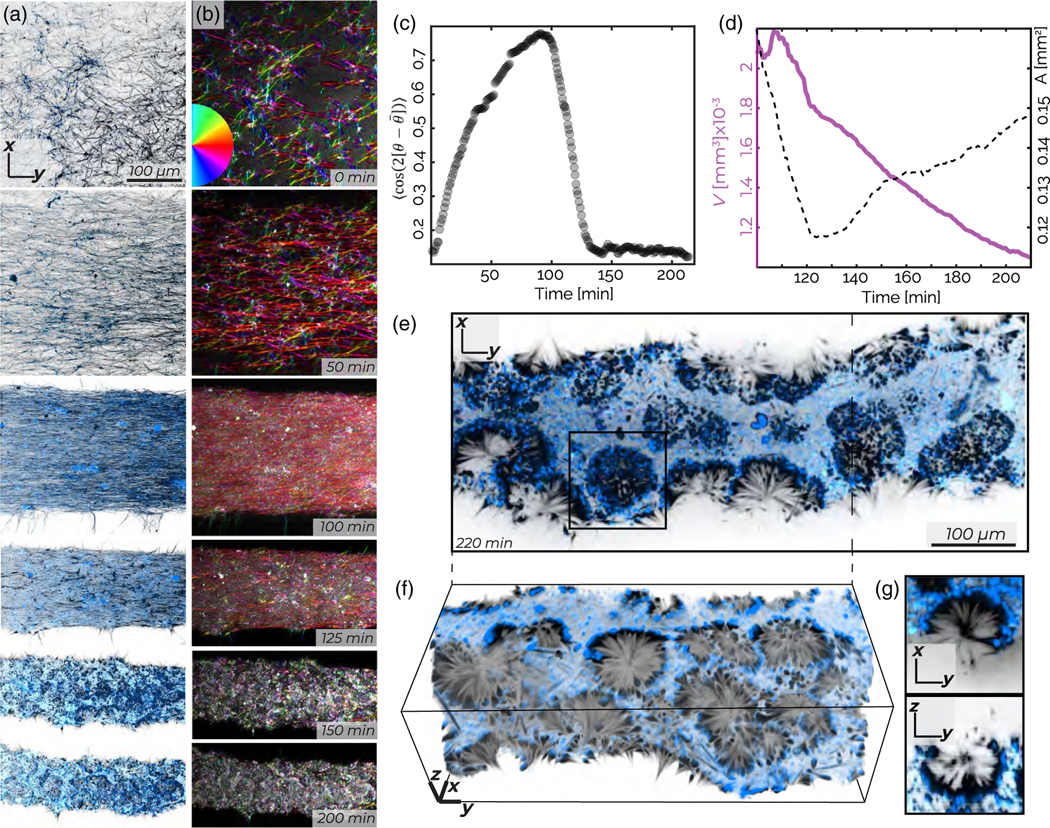
Contracting gel: Contractions yield nematic alignment and surface roughening. (a) *z*-projected images demonstrate that decreasing network volume leads to increasing nematic alignment. (b) *z* projection of the microtubule nematic order. Hue indicates the nematic director indicated by the color wheel, while intensity indicates coherency (Supplemental Material [32]). (c) The microtubule nematic order parameter increases during contraction and then decreases during roughening. (d) The contracting network’s volume (solid purple line) decreases continuously. Its surface area (dashed black line) initially decreases but then increases. (e) A 10 *μ*m *z* projection of the material after surface roughening generates spherical cavities. (f) A cropped 3D projection highlights the invaginated structure of the microtubule network. (g) *x-y* and *z-y* show a hemispherical cavity. The sample is composed of 10 *μ*M tubulin (black) and 200 nM kinesin (blue).

Samples prepared with even higher tubulin concentrations (10 *μ*M) also undergo global contractions but exhibit a distinct kinetic pathway and a different final structure from the above-described bilayers. The sample evolution proceeds in two stages: an initial global contraction followed by morphological surface roughening (Video S4). In the first stage, the initially isotropic network develops nematic order while contracting [Figs. 4(a), S2, and S3]. We define *θ* as the local orientation of microtubule bundles in the structure’s interior and $\bar{\theta }$ as the average bundle orientation [Fig. 4(b), Supplemental Material [32] ]. The scalar order parameter $S=\langle \cos(2[\theta -\bar{\theta }])\rangle$ indicates the degree of nematic ordering, with 0 representing isotropic structure and 1 representing perfect alignment (Supplemental Material [32]). As the network contracts, its volume *V* decreases monotonically, while the order parameter *S* of the enclosed microtubules increases [Fig. 4(c)].

After approximately 120 min, the heretofore increasing nematic order parameter *S* starts decreasing sharply, signaling the onset of the second stage [Fig. 4(c)]. Simultaneously, the network surface area *A*, which had previously fallen by a factor of 2, begins to increase [Fig. 4(d)]. This transition is concomitant with morphological changes, in which the smooth interface of the contracting network starts roughening. Surface roughening is accompanied by the formation of a dense monolayer consisting of a kinesin sheet with outwardly pointing microtubules, which envelopes the contracting network [Fig. 4(e)]. Over time, the roughening surface develops invaginations that rearrange into hemispherical cavities with radii of approximately 25–50 *μ*m [Figs. 4(e) and 4(f)]. Microtubules protruding from the surfaces of the hemispherical cavities reach the cavities’ center, thus creating inverted asters with a sheet of kinesin half-enveloping radially splayed microtubules [Fig. 4(g)]. While forming hemispherical cavities, the active dynamics of these samples cease, possibly due to the exhaustion of PEP in the ATP regeneration system.

**FIG. 5.**
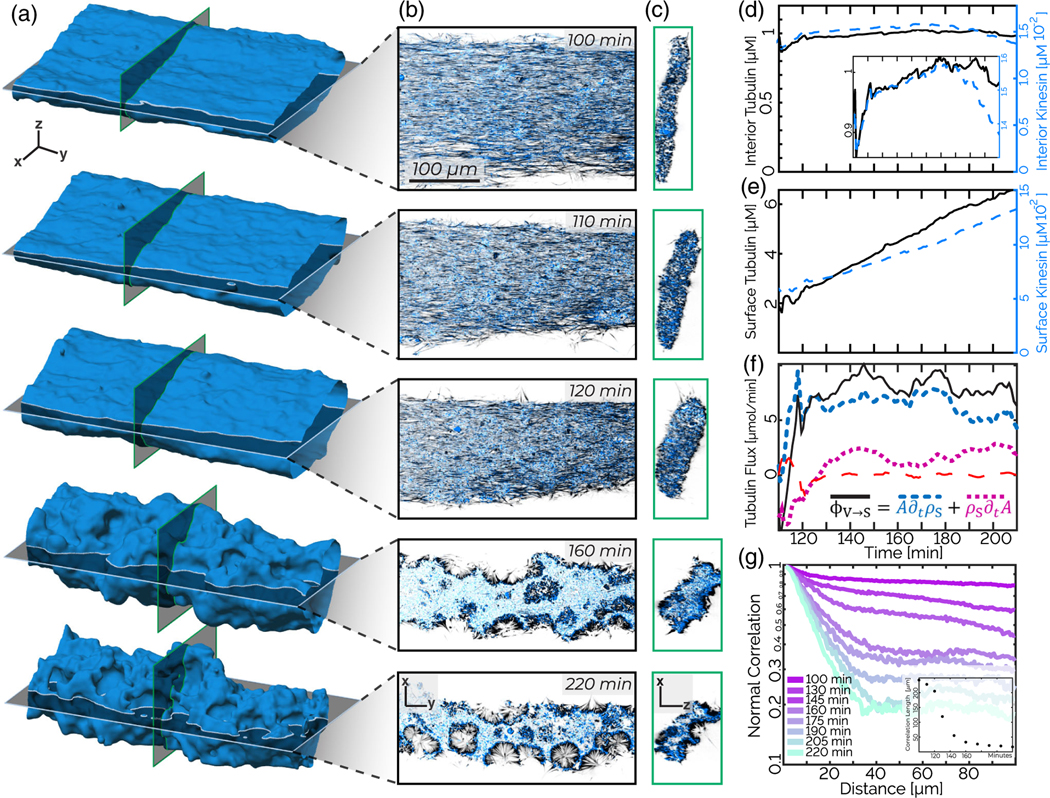
Contracting gel: Surface roughening is accompanied by the formation of a surface-bound monolayer. (a) Time series of a surface of a contracting network. (b) *x-y* slices of data corresponding to cuts shown in the previous panel reveal the formation of a monolayer and invaginations at late times. (c) *x-z* slices show a contracting cross section until the roughening commences. (d) Tubulin and kinesin density within the interior of the contracting network is constant during the roughening phase. (e) Tubulin and kinesin density within 5 *μ*m of the surface increases during the roughening phase. (f) The flux of microtubules from the interior to the surface ${\text{Φ}}_{V\to S}$ (black solid line), the microtubule surface density $A\partial_{t}\rho_{s}$ (blue dashed line), and the change in surface area $\rho_{s}\partial_{t}A$ (purple short-dashed line) as a function of time. The red long-dashed line indicates the sum of all three terms. (g) Normal-normal spatial correlations show faster decay as the material roughens. These correlations are calculated only on a bisected surface, to reduce the influence of the overall surface curvature. Inset: Exponential fits to the normal-normal correlation decay between 10 and 20 *μ*m show correlation length decreases by 200 *μ*m over 50 min. The sample consists of 10 *μ*M tubulin (black) and 200 nM kinesin (blue).

We reconstruct the network 3D structure using a morphological snake level set algorithm [Figs. 5(a) and 5(b), Supplemental Material [32]] [39–41]. The surface and cross-sectional views show an initial rounding of the network cross section, followed by a subsequent roughening [Figs. 5(c), S4, and S5]. Numerical representation of the contracting network allows us to quantify the distribution of the cytoskeletal material both on the surface and within the interior of the contracting network. During the second stage, while the density of the interior protein remains nearly constant [Fig. 5(d)], the density of kinesin-4 and microtubules within 5 *μ*m of the surface increase threefold [Fig. 5(e)].

To understand whether the protein-dense shell arises simply from geometric deformation of the surface or by drawing material from the bulk, we quantify the kinematics of the partitioning between the dense network surface and its contracting interior. In the roughening stage, the surface area A increases [Fig. 4(d)]. In the absence of any material flux between the surface and the interior, the areal density of surface-bound microtubules *ρ*_*S*_ decreases proportionally to the surface area growth: $A\partial_{t}\langle \rho_{S}\rangle =-\langle \rho_{S}\rangle \partial_{t}A$ (Supplemental Material [32]). We find that these two terms are, in fact, far from equal and opposite [Fig. 5(f)], suggesting that there is substantial flux from the interior to the surface. Meanwhile, the sum total of all microtubule fluorescence is constant. The implied mass conservation is described by

(2) $$A\partial_{t}\langle \rho_{S}\rangle +\langle \rho_{S}\rangle \partial_{t}A={\text{Φ}}_{V\to S},$$

where ${\text{Φ}}_{V\to S}$ is the flux of material from the interior to the surface. We then independently measure the flux of microtubules leaving the interior of the contracting network:

(3) $${\text{Φ}}_{V\to S}=-V\partial_{t}\langle \rho_{V}\rangle -\langle \rho_{V}\rangle \partial_{t}V,$$

Where $\langle \rho_{V}\rangle$ is the average volumetric density of microtubules and *V* is the volume of the interior, and find that it quantitatively accounts for the increasing density of the surface-bound microtubules $A\partial_{t}\langle \rho_{S}\rangle$ [Fig.5(f)]. Our analysis reveals that the density change due to surface area increase $\langle \rho_{S}\rangle \partial_{t}A$ is small compared to the mass transfer due to the flux from the interior to the surface ${\text{Φ}}_{V\to S}$. The mechanism that drives the flux of microtubule transport from the interior to the surface remains unknown.

To quantify the roughening transition, we measure the spatial correlations of the surface normals (Fig. S9). A normal vector $\hat{n}(r,t)$ describes the network at each surface point *r* at time *t* (Supplemental Material [32]). The averaged correlation between all normal vectors, separated by a geodesic of length Λ, is given by

(4) $$C(\text{Λ},t)=\frac{\langle \hat{n}(r,t)\cdot \hat{n}(r+\text{Λ},t)\rangle }{\langle \hat{n}(r,t)\cdot \hat{n}(r,t)\rangle },$$

where angular brackets indicate a spatial average over all initial points and all geodesic paths of length Λ. At the beginning of the roughening stage, the network has an extended flat shape which reflects the chamber geometry. When restricted to either the top or bottom of the surface, pairs of normal vectors $\hat{n}$ point in similar directions even at large distances. Consequently, *C*(Λ, *t*) remains close to unity for all values of Λ. As the surface roughens with time, the correlation between surface normals $\hat{n}$ decreases. *C*(Λ, *t*) develops a plateau at large distances, where the plateau magnitude decreases with time [Fig. 5(g)]. At smaller length scales, ranging from 1 to 30 *μ*m, *C*(Λ, *t*) exhibits exponential decay. The rate of the exponential increases sixfold from the beginning to the end of the roughening process. The long-range normal-normal correlation decays from *C*(40 *μ*m;100 min) ≈0.85 to *C*(40 *μ*m;220 min) ≈0.2.

### D. Active foam: Splaylike deformations, self-tearing, and roughening yields an active foam

**FIG. 6.**
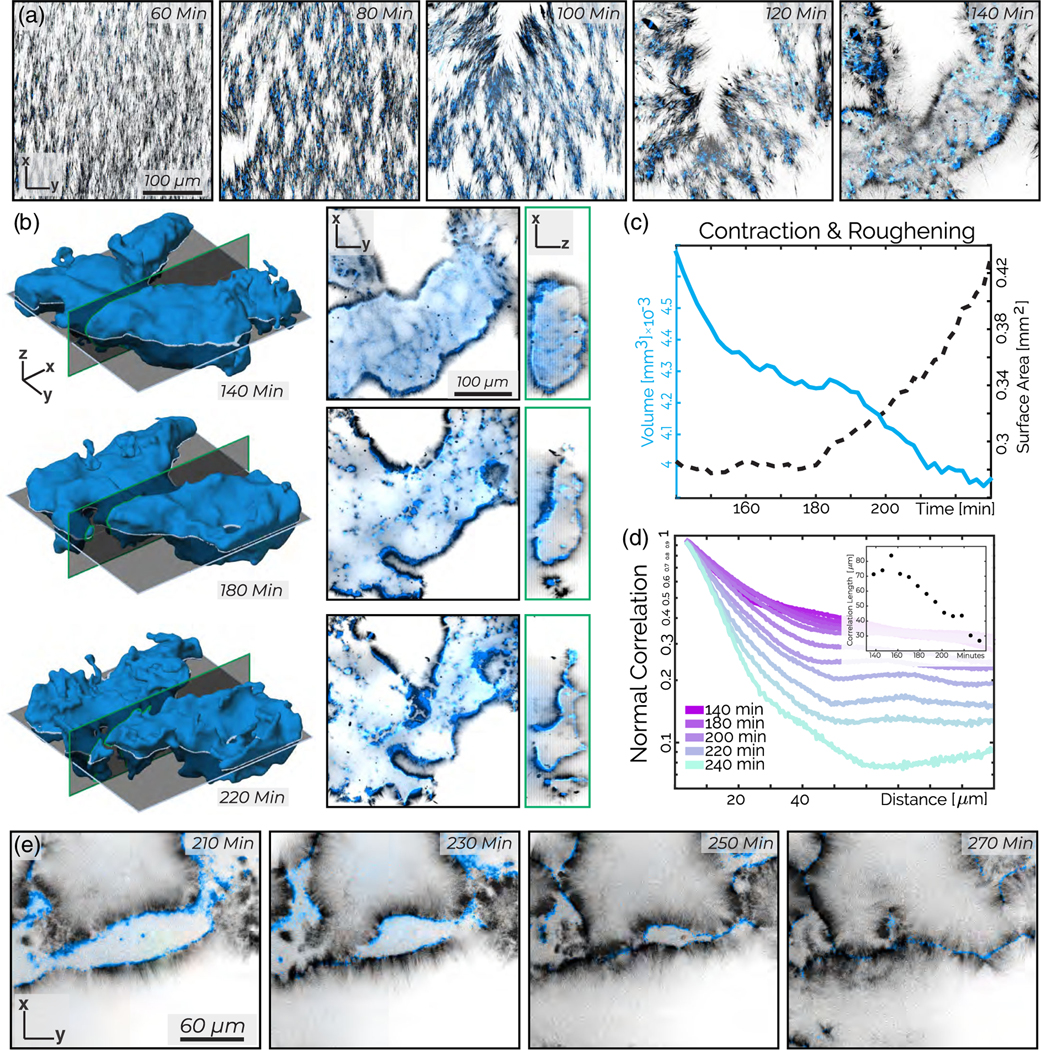
Active foam: splaylike deformations, self-tearing, and roughening at the highest microtubule concentrations. (a) Maximum intensity *z* projections over 3 *μ*m show a splaylike instability that generates density variation and self-tearing that yields condensates. (b) Evolution of a contracting condensate surface (left) *x-y* and *x-z* image cross sections (right). (c) The volume (solid blue curve) and surface area (black dashed curve) of a contracting condensate as a function of time. (d) The spatial correlation between surface normal vectors decay over time. Inset: Exponential fits to the normal-normal correlation decay between 5 and 20 *μ*m show correlation length decreases by 50 *μ*m over 80 min. (e) Two surface-bound monolayers zippering into a bilayer. The sample contains 200 nM kinesin (blue) and 40 *μ*M tubulin (black).

At the highest tubulin concentrations studied (40 *μ*M), we observe a multistage kinetic pathway which has both similarities and differences with the intermediate regime (Video S5). The microtubules have an initial orientational order even before the onset of contractions. Such a state exhibits subtle bend deformations, which are a signature of extensile stresses (Fig. S11) [42]. However, the buckling dynamics quickly transition into more dramatic splaylike deformations, the onset of which breaks up the continuous network by generating sharp density variations between filament-rich and filament-poor regions [Fig. 6(a), 80 min]. These changes in orientational order and local density fluctuations yield finite-sized condensates that are well separated from a background fluid mostly devoid of protein [Fig. 6(a), 140 min]. A high-density monolayer of kinesin and microtubules envelopes the condensate surface, with microtubules aligned along the surface normal. The monolayer-covered condensates are similar to those observed at lower filament concentrations. The main difference is that active stresses rupture the network, creating finite-sized structures. In contrast, lower microtubule concentrations generate only one contracting network, which does not break apart.

After their formation, condensates exhibit surface roughening. Using the previously described algorithm, we numerically generate surfaces describing the evolution of the condensate morphology [Fig. 6(b)]. The condensate’s volume decreases continuously, while its surface area A remains constant until approximately 160 min, after which A increases sharply [Figs. 6(c), S6, and S7]. As roughening continues, the mean curvature increases, and the normal-normal correlation *C*(*r*) decreases [Figs. 6(d) and S8]. High-resolution images reveal the macroscopic mechanism driving the roughening transition. Crumpling monolayers encounter each other, generating a zippering transition of the kinesin decorated surfaces, which locally produces a well-defined bilayer [Fig. 6(e)].

**FIG. 7.**
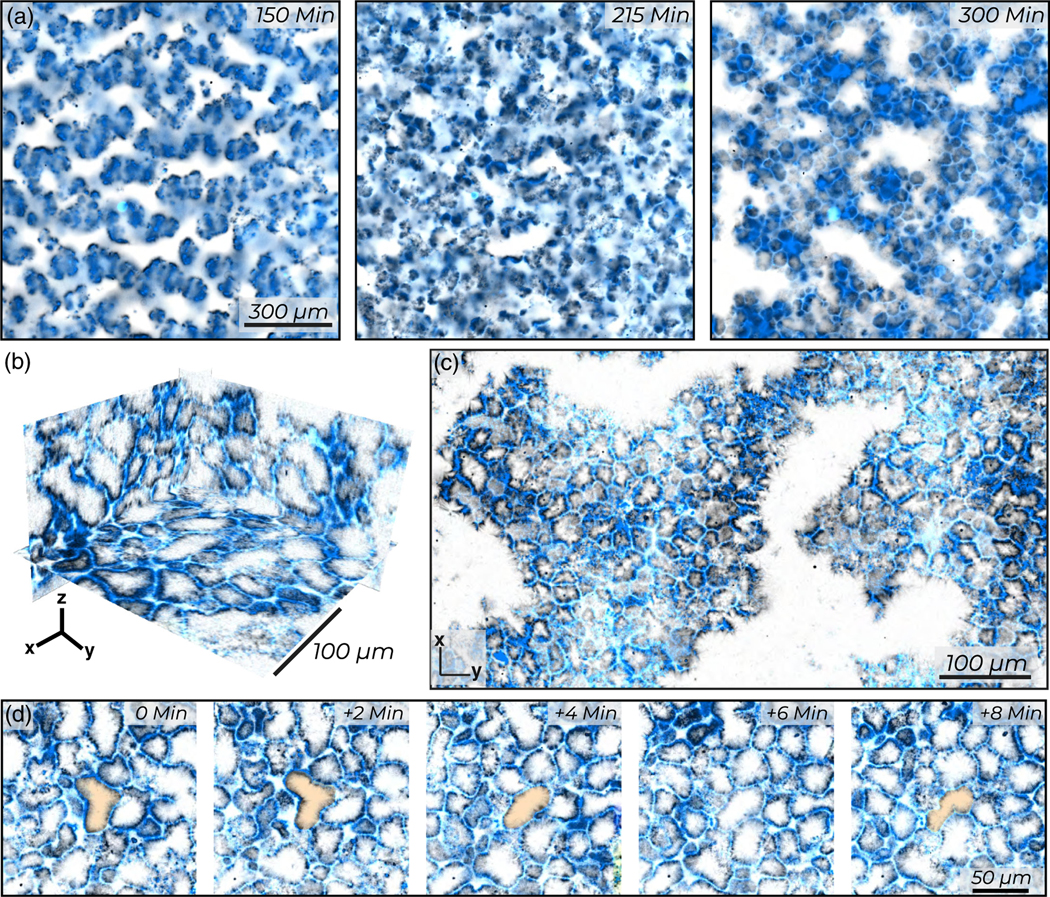
Active foam: Surface roughening yields an active foam. (a) Morphological change from monolayer envelopes to a percolated foam. (b) Ortho-slices show the complex 3D structure of the active foam. (c) Maximum intensity *z* projection over 10 *μ*m illustrates distinct foam cells which can have free ends or open faces. (d) A foam cell undergoes topological rearrangements in an active foam. Samples are constituted from 200 nM kinesin (blue) and 40 *μ*M tubulin (black).

On long times, the surface roughening transition generates an active foam, a distinct active state consisting of a 3D network of bilayers that connect through junctions [Figs. 7(a) and S11]. As in conventional foam, the interconnected bilayer surfaces form cells [Figs. 7(b) and S12]. Unlike conventional foams, cells in an active foam have elongated or even winding shapes, while the constituent bilayers have free-standing edges [Figs. 7(c) and S12(b)]. The borders of the active foam compartments consist of microtubule/kinesin-4 bilayers [Figs. 7(b) and 7(c)]. The active foam exhibits topological rearrangements. Individual cells deform, while bilayer walls move to change the local topology [Fig.7(d), Video S6]. Thus, the surface roughening transition is the first stage of a unique morphological transition in which a continuous and smooth space-filling condensate transforms into perforated foamlike structures. The development of an active foam and its rearrangements remains an important topic for future investigations.

### E. Extensile fluid: A bundling-induced transition from contraction to extensile gels

**FIG. 8.**
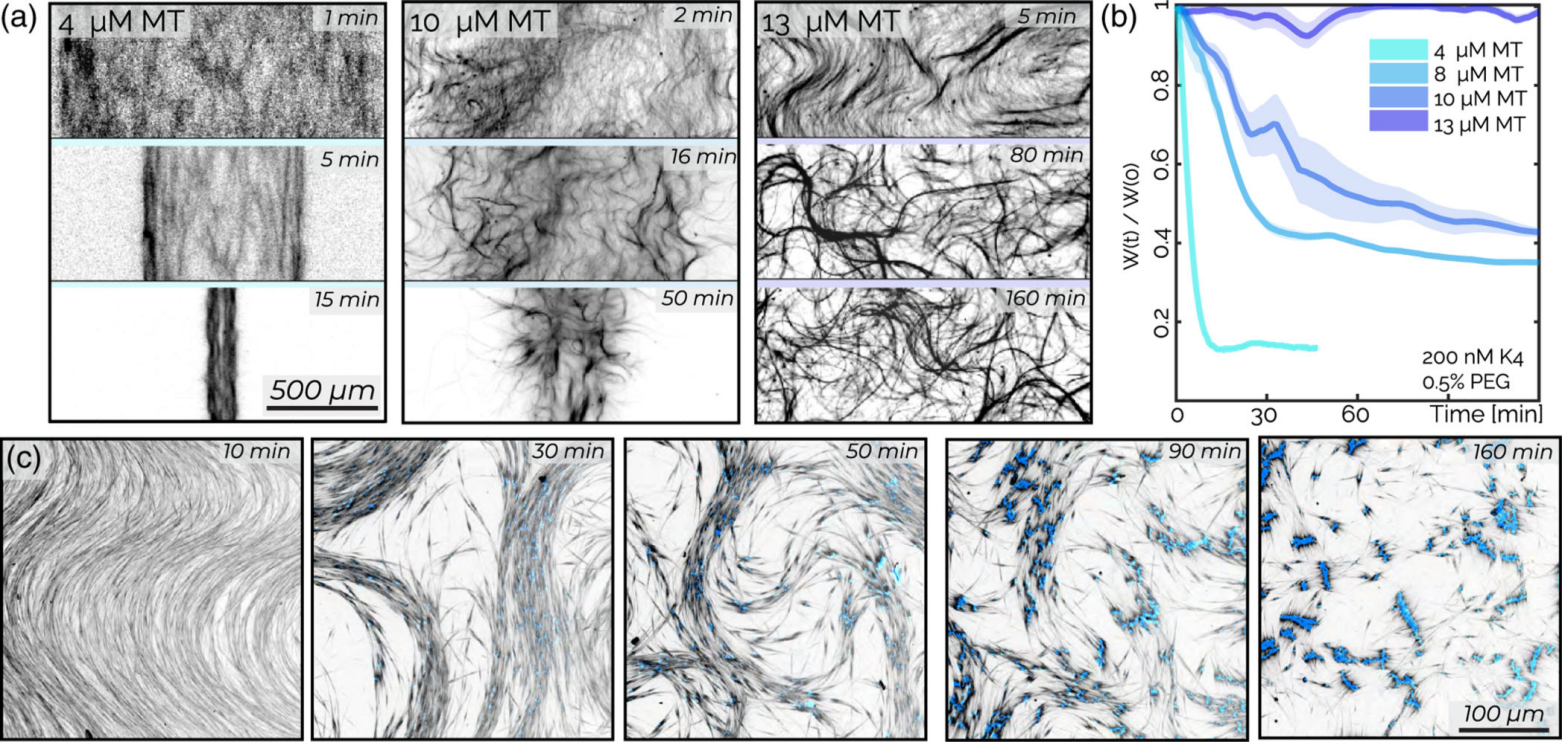
Extensile fluid: a bundling-induced transition from contraction to extensile gels. (a) The evolution of the shear-aligned microtubule network depends on filament concentrations. Samples have 0.5% PEG, 200 nM kinesin. (b) The average microtubule network width *W*(*t*), normalized by the initial width *W*(0), decreases over time, with lower microtubule densities contracting faster. The shaded region indicates the standard deviation from data taken at five nonoverlapping positions over the long axis of the chamber. (c) Extensile instability leads to the formation of a bilayer structure. This sample chamber is 30 *μ*m thick; this sample contains 100 nM kinesin (blue), 80 *μ*M tubulin (black), and 0.1% PEG.

So far, we described kinesin-4-driven microphase separation and associated local and global contractions which occur with increasing microtubule concentrations. In contrast, conventional kinesin-1 generates extensile stresses in the presence of a microtubule bundling agent [13,43]. To investigate the capability of kinesin-4 motors to generate extensile stresses, we add a nonadsorbing polymer, polyethylene glycol (PEG), which bundles microtubules while still allowing for their relative motor-driven sliding [44]. At low microtubule concentrations (4 *μ*M), global contractions occur even in the presence of 0.5% w/w PEG [Fig. 8(a)]. Beyond a critical filament concentration (10 *μ*M tubulin), the material exhibits initial self-generated bendlike patterns which are suggestive of extensile stresses (Video S7) [1,45]. On longer timescales, these materials do not contract but rather yield a continuously rearranging network, similar to those previously studied [Fig. 8(a)] [46,47].

The contractile to extensile transition is quantified by plotting the network width as a function of time *W*(*t*) [Fig. 8(b)]. At low filament concentrations, *W*(*t*) monotonically decreases and then plateaus, characteristic of contraction. Further increasing microtubule concentration results in a network that spans the entire chamber while continuously rearranging. Therefore, *W*(*t*) does not change over time. Using particle image velocimetry, we find that the mean microtubule network speed increases with increasing kinesin concentration. In contrast to kinesin-1 studies, increasing kinesin-4 concentration increases the velocity-velocity correlation length scale (Fig. S10) [47].

We also observe that extensile gels could transform into globally contracted bilayers [Fig. 8(c), Video S8]. Upon preparation, an active mixture (0.1%−0.3% w/w PEG, 80–90 *μ*M tubulin) exhibits a bend instability and fluidizes. However, on longer timescales, distinct segments of kinesin-4 appear. As these segments become prominent, the motor-driven dynamics slows down. This dynamical transition is concomitant with the appearance of local bilayerlike arrangements. In these bilayers, kinesin-4 forms a central line with microtubules pointing outward on both sides.

### F. A nonequilibrium phase diagram of kinesin-4 and microtubules

**FIG. 9.**
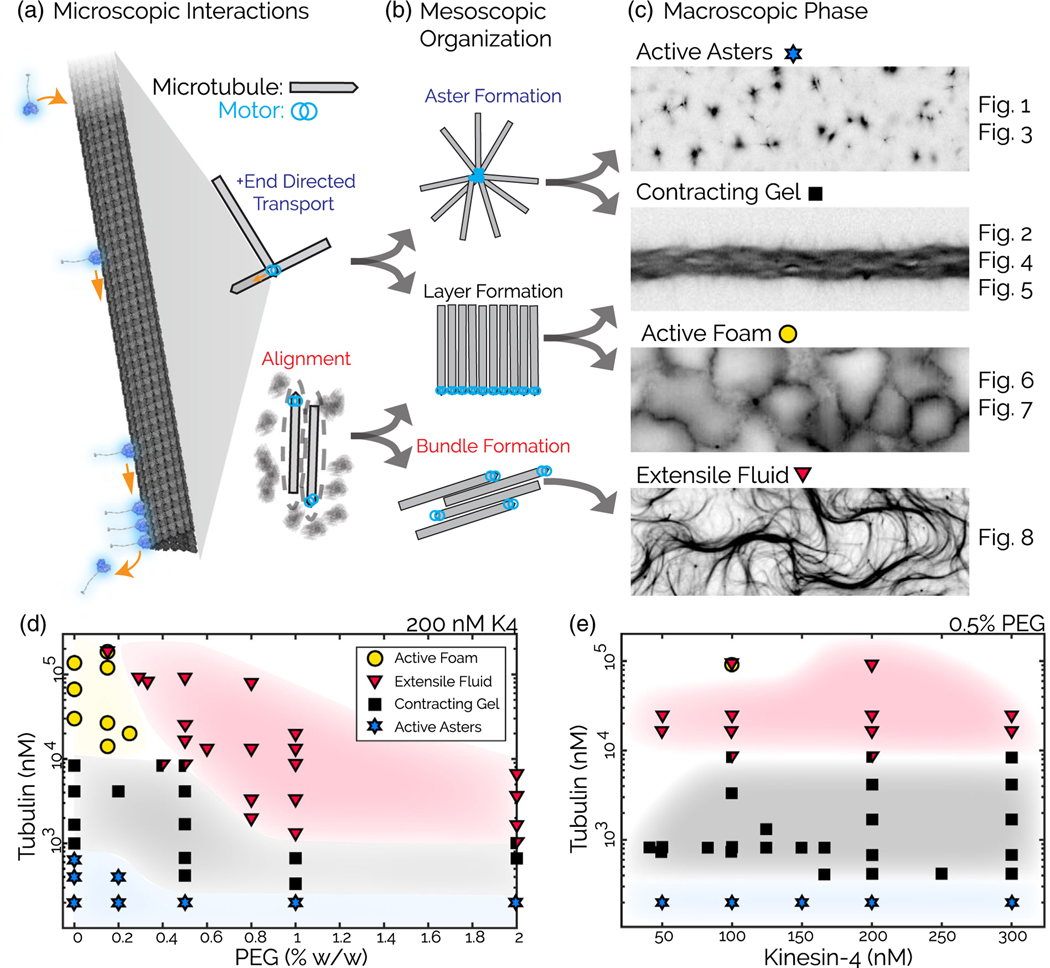
A nonequilibrium phase diagram of kinesin-4 and microtubules. (a) Microscopic building blocks: Kinesin-4 (blue) attaches to a microtubule (gray), walks to the microtubule plus end, and accumulates at the plus end, creating a heterogeneous filament that can interact with other filaments by directed transport or via steric alignment induced by PEG. (b) Mesoscale organizational motifs include asters, layers, or bundles. (c) Hierarchically organized mesoscale building blocks yield macroscopic phases including dynamic asters, globally contracting gels, active bilayer foams, and fluidized extensile bundles. (d) Phase diagram at 200 nM kinesin as a function of tubulin and PEG concentration. (e) Phase diagram at 0.5% PEG (w/w) as a function of protein concentrations.

A one-dimensional sweep of tubulin concentration in the absence of PEG yields active microphase separated phases, while adding PEG produces an active extensile fluid. To further characterize the system, we map the nonequilibrium phase diagram by creating samples between 50 and 300 nM kinesin-4, 0.2–180 *µ*m tubulin, and 0%–2% PEG [Figs. 9(d) and 9(e)]. At the lowest microtubule concentrations, the active material contracts into localized asters over a wide range of PEG and kinesin-4 concentrations. Increasing microtubule concentration generates global contractions, again over a wide range of PEG and kinesin-4 concentrations. At the highest microtubule concentrations, with little or no PEG, we observe active foams. Adding PEG in this regime transforms active foams into extensile turbulentlike gels similar to those seen in kinesin-1-driven systems.

Presumably, introducing PEG suppresses the formation of asters and bilayer foams while promoting the formation of bundles that generate extensile dynamics [Fig. 9(d)]. Kinesin-4 concentration determines the speed of the autonomous dynamics but does not substantially affect the boundaries between the extensile and contracting phases [Fig. 9(e), Supplemental Material [32]]. The long-term nonequilibrium phase behavior described here depends on the initial and boundary conditions and the sample history (Fig. S13).

## III. DISCUSSION

In cytoskeletal active matter, extensile active stresses drive continuous turbulentlike flows, while isotropic contracting active stresses generate local or global collapse [13,16,22,38,48–52]. We study the self-organization of microtubules and kinesin-4, a tip-accumulating molecular motor. High filament concentrations and bundling agents generate extensile turbulent flows. Reducing either the microtubule or PEG concentrations results in contraction. These observations demonstrate that the form of the active stress is not solely dictated by the molecular properties of cytoskeletal components but is also dependent on their concentration. This insight is valuable for relating the mesoscopic active stresses to the structure, interactions, and dynamics of the microscopic constituents [20,53–57]. In the contracting regime, we observe a myriad of active microphase separated structures. The lowest filament concentration sample yields isolated asters (Fig. 1). With increasing filament concentrations, asters transform into 1D wormlike structures, extended 2D bilayers, and foamlike 3D material (Figs. 2 and 7). Taken together, our findings have several implications for our understanding of cytoskeletal active matter.

Asterlike structures are observed in mixtures of microtubules and various molecular motors [2,16,58–61]. Theoretical models of such asters are sometimes couched in the language of topological defects in liquid crystals. However, the asters studied here are well-isolated structures in a filament-free background fluid; thus, they are more reminiscent of equilibrium amphiphile-based micelles. Instead of hydrophobic interactions, their condensation is driven by tip-accumulating molecular motors. With increasing concentration, amphiphilic systems form 1D wormlike micelles, 2D membranes, and space-filling 3D lamellar, hexagonal, or disordered gyroid phases [25]. We observe active analogs of these higher-order phases. Once the microphase separation is complete, motors continue to reconfigure the material, as we observe for both wormlike structures and active foams (Videos S1, S3, and S6). Kinesin-4 drives these large-scale events by generating active stresses that are likely distinct from those postulated for a suspension of aligned active filaments.

Molecular motors can mediate different filament interactions. For example, they can drive inter-filament sliding within an aligned bundle, or they can cluster tips of isotropically arranged filaments [16,28,55]. Clusters of kinesin-1 motors are thought to primarily induce filament sliding [55]. However, observation of asters in such systems suggests that they retain a small degree of end binding [59]. In comparison, kinesin-4 has an enhanced end-binding property, which has been characterized on the single-filament level [28,31]. We develop a model of aster structure that predicts the microtubule profile from a given kinesin profile, but it does not explain the size of the kinesin core. The latter is likely related to the size of the kinesin-4 cap. More experimentation is needed to elucidate this point, as single-filament experiments suggest that the cap size depends on protein concentrations and microtubule length [31]. The motility of the kinesin themselves might also determine the kinesin-4 cap length. Notably, theoretical models accounting for asymmetric motor distributions or tip-anchoring predict structures that are qualitatively similar to the active asters and foams [62,63]. In general, the balance of spatial filament decoration and interfilament sliding by molecular motors might determine the range of possible phases of an active cytoskeletal material and is a promising avenue for further investigation.

Active microphase separation has relevance to biological systems. The self-organization of microtubules and molecular motors has been studied in *Xenopus* egg extracts [64,65]. Dynein drives aster assembly in *Xenopus* egg extracts, which globally contract at higher filament concentrations [38,66,67]. Such asters are used as models for spindle pole assembly [67]. Under other conditions, stabilized microtubules in *Xenopus* egg extracts assemble into structures reminiscent of the bilayers observed in the present work [30]. In these experiments, extended bilayers of taxol stabilized microtubules form, with their minus ends pointing away from the midplane. These bilayer structures serve as models for the spindle midzone, the array of microtubules that assembles between segregating chromosomes and drives the spindle elongation and chromosome separation [68–70]. Much prior work on spindle midzones focuses on factors that determine the extent of antiparallel overlap of the microtubule ends [28,71]. However, the reason why this narrow region of antiparallel overlap stays well aligned across the entire spindle width remains poorly understood. The similarity between the bilayers observed in the present work, those formed in *Xenopus* egg extracts, and the spindle midzone itself suggests that similar principles might govern the self-organization of all of these structures.

Besides revealing a range of active microphase states, our work also demonstrates rich kinetic pathways that lead to the formation of these phases. These pathways are influenced by the interplay between the tendency of rodlike filaments to align due to excluded volume interactions and the propensity of tip-adhering kinesin motors to drive microphase separation. We observe filament alignment at high microtubule concentrations, which either occurs initially during sample loading or develops over time in a contracting network (Figs. 4 and S11). Theory dictates that aligned active filaments are inherently unstable [42]. Specifically, extensile active stresses drive the bend instability as we observe for the kinesin-4 system in the presence of bundling interactions (Fig. 8) [46,72]. Analogously, contractile systems exhibit splay instabilities, but these have not been studied experimentally.

At high microtubule concentrations, samples exhibit both aligned filaments and network contraction (Figs. 4 and 6). Thus, they are a good candidate for observing splay instability. Indeed, we observe splaylike deformations, but these are associated with self-tearing. This might be a consequence of the extended nature of microtubule filaments. In polymeric liquid crystals, such as microtubule-based nematics, splay deformations generate local variations in the filament concentration [73]. Thus, splay instabilities lead to sharp density gradients, which, in turn, could lead to self-tearing, which yields finite-sized condensates. Beyond this point, the system starts exhibiting structural rearrangements that are likely driven by the tip accumulation of molecular motors. In particular, the rapidly formed condensates become enveloped by a monolayer of aligned microtubules, which are anchored to a 2D sheet of kinesin motors. The subsequent surface roughening transition is related to the zippering of monolayers into bilayers (Fig. 6). It generates dramatic topological rearrangements that transform simple compact condensates into a perforated active foam. Active foams are composed of bilayers, which have both locally aligned filaments and tip-accumulated motors. Thus, they resolve the above-described constraints that govern the dynamics of kinesin-4 and microtubule systems.

In summary, we demonstrate that kinesin-4 motors self-organize microtubules into a myriad of hierarchical structures. At a single-filament level, kinesin-4 motors accumulate at microtubule tips to define a spatially heterogeneous elemental unit capable of higher-order self-organization. This segmented structure results from a dynamical process, in contrast to amphiphilic systems, where the spatial heterogeneity of the basic building blocks is permanently programmed in the amphiphile molecular structure. Tip-decorated microtubules locally condense to generate higher-order radial asters. In turn, asters can merge to form extended bilayer sheets. At higher filament concentrations, the bilayer sheets form a tissuelike active foam that undergoes intriguing active dynamics and motor-driven topological rearrangements. Taken together, these results demonstrate a distinct category of active microphase separation. Relating these diverse large-scale behaviors to the molecular properties of the constituent kinesin motors and microtubules poses a significant theoretical challenge.

## Supplementary Material

Video 7

Supplementary

Video 8

Video 5

Video 4

Video 3

Video 2

Video 1

Video 6

## APPENDIX: METHODS

### 1. Sample preparation

We study kinesin-4-driven dynamics by combining GFP-labeled kinesin with Alexa-647 labeled stabilized microtubules in a buffered solution with an ATP regeneration system. The solution consisted of de-ionized (DI) water with 80 mM PIPES (piperazine-*N*, *N*′-bis), 5 mM magnesium chloride, 1 mM egtazic acid, and 1.4 mM ATP (adenosine triphosphate, Sigma A2383). To prevent the ATP concentration from changing, we include an ATP regeneration system of 52 mM PEP (phosphoenolpyruvate, Alfa Aesar B20358) and 0.034% pyruvate kinase (PK/LDH, Sigma P-0294). To prevent photobleaching, we add 4.2 mM DTT (dithiothreitol, ACROS Organics 16568), 2.5 mg/mL glucose (Sigma G7528), 0.03 mg/mL catalase (Sigma C40), and 0.17 mg/mL glucose oxidase (Sigma G2133). This solution is adjusted to a *p*H of 6.8 with potassium hydroxide. When noted, experiments include 35 kDa PEG (polyethylene glycol).

The full-length human kinesin-4 clone Kif4A or fluorescent Kif4A-GFP are expressed in sf9 cells as described previously [27]. We purify tubulin from bovine brains according to a previously published protocol [74]. This tubulin is polymerized and stabilized into microtubules by mixing 60 uM tubulin with 3 mM of the nonhydrolyzable GTP analog GMPcPP (guanosine-5-[(*α*,*β*)-methyleno]triphosphate, Jena Biosciences NU-405), and a solution of 1 mM DTT, 80 mM PIPES, 2 mM magnesium chloride, and 1 mM egtazic acid in DI water adjusted to a *p*H of 6.8 with potassium hydroxide. 3% of tubulin monomers are labeled with a fluorescent dye, Alexa-Fluor 647 (Invitrogen, A-20006), by a succinimidyl ester linker according to a previously published protocol [75]. The solution is incubated in a water bath at 310 K for one hour and then left to cool to room temperature for 6 h. Polymerized microtubules are flash-frozen in liquid and subsequently thawed before creating a sample.

While all active materials consist of GMPcPP polymerized microtubules, the concentrations refer to tubulin concentrations. A microtubule consists of a repeating lattice of approximately 13 tubulin monomers, and each ring of the lattice is 4 nm [76]. Thus, if the mean microtubule length is approximately 4.9 *μ*m, each microtubule has roughly 16 000 tubulin monomers.

### 2. Chamber preparation

Each experiment occurs in a chamber with dimensions of 1.5 mm × 0.1 mm × 18 mm unless noted otherwise. The chamber consists of a glass top and bottom, with parafilm spacers sealed with NOA 81 UV adhesive (Norland Products, 8101) at both ends. The glass is coated with a polyacrylamide brush to suppress protein adsorption onto the glass [77]. To bond parafilm to the glass, we warm the parafilm to 338 K and press it onto the glass with the rounded end of a microcentrifuge tube. This process leads to chambers that are 80–100 *μ*m in height.

### 3. Microtubule length distribution measurements

To measure microtubule length distributions, we flow dilute microtubules into an untreated glass chamber. Microtubules adsorbed onto the glass are imaged with a 100× objective with a 1.2 NA (numerical aperture) and an automated stage. The resulting dataset is segmented based on a simple threshold. Each segmented object is then fit to an ellipse. If the ellipse has a thin minor axis compared to its principal axis, then it is recorded as a microtubule with the principal axis as the length. This process discards overlapping or out-of-focus microtubules.

### 4. Microscopy

Fluorescence images are captured using a Nikon Ti2 base attached to an Andor Zyla using a 4× Nikon Plan Apo Lambda (NA 0.2) objective or a 10× Nikon Plan Fluor objective (NA 0.3).

Confocal microscopy images are captured with a Crest X-Light V2 spinning disk system attached to a Nikon Ti2 base and a Hamamatsu ORCA-Flash4.0 V3. The objective used for the aster sedimentation data is a 40× Plan Fluor objective (NA 0.75). The objective used for all other data is a 40× Apo long working distance water immersion objective (NA 1.15). Zeiss Immersol W, an NA matched oil substitute, prevents imaging deterioration due to water evaporation during long acquisitions.
