# Supplementary material for: Active Microphase Separation in Mixtures of Microtubules and Tip-Accumulating Molecular Motors
Source: Phys Rev X. Author manuscript; Available in PMC 2023 Jan 12. (PMC9835929; doi:10.1103/physrevx.12.031006)
Supplement: Supplementary [file NIHMS1852678-supplement-Supplementary.pdf]

Active microphase separation in mixtures of microtubules and tip-accumulating molecular motors Supplementary Material

Bezia Lemma, Noah P. Mitchell, Radhika Subramanian, Daniel J. Needleman, and Zvonimir Dogic

I. SUPPLEMENTARY INFORMATION

A. Prediction of Aster and Bilayer Structure

We analytically match the microtubule intensity profile in asters and bilayers using only the intensity profile of molecular motors and a measured length distribution of microtubules [Fig. 1(c)]. To do this, we model the microtubule profile as arising from microtubules of various lengths attached to the kinesin by their ends. This is equivalent to the convolution of the molecular motor profile with a probability distribution of microtubule length. We represent the measured length distribution of stabilized microtubules with a log-normal distribution $f(\Lambda)$:

$$f(\Lambda) = \frac{1}{\Lambda S\sqrt{2\pi}} \exp\left(-\frac{(\ln(\Lambda) - M)^2}{2S^2}\right), \tag{S1}$$

where Λ is the non-dimensionalized length L/L_0, and M and S are fit parameters related to the dimensionless mean μ/L_0 and the variance σ^2/L_0^2 as:

$$\mu/L_0 = e^{M+\frac{S^2}{2}}, \tag{S2}$$

$$\sigma^2/L_0^2 = e^{S^2+2M}\left(e^{S^2} - 1\right). \tag{S3}$$

The probability that a microtubule has a dimensionless length greater than d/L_0 is the integral of that distribution from the length d/L_0 out to infinity:

$$I_{1d}(d) = \nu_{1d}\int_{d/L_0}^{\infty} f(\Lambda)d\Lambda = \frac{\nu_{1d}}{2} + \frac{\nu_{1d}}{2} erf\left(\frac{M - \ln(d/L_0)}{\sqrt{2}S}\right), \tag{S4}$$

where ν_{1d} is a normalization factor that includes the conversion to fluorescent intensity. This equation represents the normalized microtubule intensity profile of microtubules perpendicularly anchored on one side of a plane. The only fit parameter is the normalization factor ν_{1d}, as all other variables have been extracted from the measured length distribution.

In order to predict the structure of a radially symmetric aster, it is helpful to extend this analysis to radial coordinates. A radially oriented microtubule at a distance r from its kinesin anchor takes on the same form, but with a factor $1/r$:

$$I_r(d) = \frac{\nu_r}{2\pi r}\int_{r/L_0}^{\infty} f(\Lambda)d\Lambda = \frac{\nu_r}{4\pi r} + \frac{\nu_r}{4\pi r} erf\left(\frac{M - \ln(r/L_0)}{\sqrt{2}S}\right), \tag{S5}$$

where ν_r is a normalization factor adjusted for radial coordinates. Finally, we convolve this result with the imaging point spread function f_{ps}, measured from 50 nm fluorescent colloids.

$$I_{MT}^{aster} = I_K^{aster} * I_r * f_{ps} \tag{S6}$$

Convolving the distribution $I_r(d)$ with the radial profile of kinesin intensity I_K^{aster} and the point spread function f_{ps} creates the radial aster microtubule intensity profile I_{MT}^{aster} which closely matches the experimental microtubule intensity profile as shown in Figure 1(e).

The equivalent calculation for the contracted bilayer's microtubule profile, shown in Figure 2(f), can be reduced to a one-dimensional problem. We construct the bilayer microtubule profile as microtubules perpendicularly anchored on a plane and thus use the 1D model for I_{1d} derived earlier. Convolving I_{1d} with the z-profile of kinesin from the bilayer $I_K^{bilayer}$ in two directions, and then convolving that profile with point spread function f_{ps} creates the bilayer microtubule z-profile $I_{MT}^{bilayer}$.

B. Aster Segmentation

The aster data consists of 2-channel z-stacks with a distance of 0.65 μm between the imaging planes. The length of a pixel is also 0.65 μm. To measure the volume and aspect ratio of asters [Fig. 1(f),(g)], we segment the kinesin channel through a simple threshold. This binary data set is refined by a Chan-Vase active contour algorithm operating on the original data set.

C. Sedimentation Height

The sedimentation height h_{MT} is the height from the base of the chamber at which 1/5 of the total microtubule fluorescence is encompassed. To calculate this height we define the cumulative microtubule density function $D(h)$, an integral of material from the floor of the chamber to height h, normalized by the total material in the chamber ρ_{tot}

$$D(h) = \frac{1}{\rho_{tot}} \int_0^h \rho(y) dy. \tag{S7}$$

The sedimentation height h_{MT} is then the height at which $D(h_{MT}) = 0.2$. The density ρ_{MT} in Figure 3(e) is the mean density of all material below the height h_{MT}.

D. Orientational Order Parameter and Coherency

We calculate orientation fields from images by identifying the principal spatial derivatives using a structure tensor [72, 73]. A structure tensor $\mathbf{T}$ of two-dimensional gradients is constructed from a 3D signal intensity field I as

$$\mathbf{T} = \begin{bmatrix} \partial_x \partial_x I_{xyz} & \partial_x \partial_y I_{xyz} \\ \partial_y \partial_x I_{xyz} & \partial_y \partial_y I_{xyz} \end{bmatrix}. \tag{S8}$$

The eigenvalue λ_{min} of $\mathbf{T}$ associated with the lowest intensity variation represents the vector $\vec{v}_{min}$ along which the intensity gradients are smallest. The direction of $\vec{v}_{min}$ gives the scalar orientation field used to calculate the orientation distribution function. The coherency C [Fig. 4(b)] is defined as the difference between the tensor eigenvalues normalized by their sum:

$$C = \frac{\lambda_{max} - \lambda_{min}}{\lambda_{max} + \lambda_{min}}. \tag{S9}$$

We calculate a field of local orientations θ from the local values of $\vec{v}_{min}$. The contractions analyzed display negligible bend in their structure, so we define a single average director $\bar{\theta}$ for the entire material as the mean value of θ:

$$\bar{\theta} = \frac{1}{N} \sum_{i=1}^{N} \theta_i. \tag{S10}$$

From this we calculate the orientational order parameter S, defined as:

$$S = \langle \cos(2[\theta - \bar{\theta}]) \rangle. \tag{S11}$$

At late times, microtubule bundles appear anchored normal to the surface. We exclude in the calculation of $\bar{\theta}$ and the orientational order parameter S by using a mask. The mask is generated from a probability field P_{in} using iLastik for pixel classification [74].

E. Surface Construction

To construct numerical surfaces, we start by acquiring confocal data such that each voxel is isotropic. These voxels are classified as "inside" or "outside" the structure of interest by using iLastik to generate a probability field P_{in}. Then a binary field F is generated from P_{in} using a morphological snake method. Next a polygonal surface S is constructed from F using a marching cubes algorithm. Finally, the surface S is remeshed at a specified triangle size using Meshlab [75]. Code for this process is available upon request.

F. Normal-normal correlation $C(r)$

To determine the normal-normal correlation of a structure we first generate a surface for that structure as described above. We then bisect the surface along the smallest moment of the material. This bisection is to exclude anticorrelations in $C(r)$ due to the curvature of the surface. We calculate a normal vector $\hat{n}(r,t)$ at each point r on the two surface halves at time t. The normal-normal correlation is calculated as

$$C(\Lambda,t) = \frac{\langle \hat{n}(r,t)\cdot\hat{n}(r+\Lambda,t)\rangle}{\langle \hat{n}(r,t)\cdot\hat{n}(r,t)\rangle} = \frac{1}{N_i N_\Lambda}\sum_i^{N_i}\sum_\Lambda^{N_\Lambda}\frac{\hat{n}(r_i,t)\cdot\hat{n}(r_i+\Lambda,t)}{\hat{n}(r_i,t)\cdot\hat{n}(r_i,t)}, \tag{S12}$$

where angular brackets indicate a spatial average over all initial points i and all geodesic paths Λ. We calculate geodesics on each half of the surface via a fast-marching mesh algorithm. Figure S9 shows the geodesic distance from a point along a contracted surface and the normal vectors of that contracted surface. Binning by lengths of the path Λ at a particular time t, we calculate $C(r)$. At small length scales, the normal-normal correlation is reasonably well fit by an exponential. The correlation length [Fig. 5(g), Fig. 6(d)] is defined as the inverse of the exponent to this fit. Code to generate normals and calculate geodesic distances is available upon request.

G. Contraction Kinematics

If the mass of proteins is conserved, there are constraints relating shape change with protein flux. We consider an enclosed network with a volume V and a boundary of area A. The total mass M is the sum of the areal surface density ρ_A, plus the sum of the volumetric density ρ_V over the volume:

$$M = \int_A \rho_S dA + \int_V \rho_V dV. \tag{S13}$$

Assuming mass conservation, the time derivative of this quantity is zero. That is,

$$0 = \partial_t M = \int_A (\partial_t\rho_A)dA + \langle\rho_A\rangle\partial_t A + \int_V (\partial_t\rho_V)dV + \langle\rho_V\rangle\partial_t V, \tag{S14}$$

where angular brackets indicate a spatial average. Given that protein is found only on the surface and in the bulk, an increase in the first two terms would signal a flux of material from the bulk to the surface, whereas an increase in the second two terms would signal a flux of material into the bulk. That is, the net flux of protein from the bulk V to the surface S is

$$\Phi_{V\to S} = A\partial_t\langle\rho_A\rangle + \langle\rho_A\rangle\partial_t A \tag{S15}$$

while the net flux of protein from the surface to the bulk is

$$\Phi_{S\to V} = V\partial_t\langle\rho_V\rangle + \langle\rho_V\rangle\partial_t V. \tag{S16}$$

H. Mean Network Speed and Velocity Correlation Length of K4 Driven Gels

The velocity field, $v(r,t)$, of the extensile fluid phase was calculated using the velocimetry package PIVLab [Fig. S10(a),(b)] [76]. From this data we calculated the the mean network speed $\langle|V|\rangle$ defined as

$$\langle|V|\rangle = \frac{1}{T_f - T_i}\sum_{t=T_i}^{T_f}\langle v(r,t)\rangle \tag{S17}$$

where T_f is the final time, and T_i indicates time shortly after the initial gel buckling instability. The average inside the sum is over space as defined by the variable r. Titrating over kinesin concentration, we found that the mean microtubule network speed $\langle|V|\rangle$ increased with kinesin concentration [Fig S10(c)].

We used the velocity field $v(r,t)$ to generate a spatial velocity-velocity correlation $A_{vel}(r)$ defined as

$$A_{vel}(r) = \frac{1}{T}\sum_t^T\langle A(r,t)\rangle = \left\langle\frac{\langle v(r,t)\cdot v(r',t)\rangle}{\langle v(r',t)\cdot v(r',t)\rangle}\right\rangle = \frac{2}{TN(N-1)}\sum_t^T\sum_i^N\sum_{j<i}^N\frac{v(r_i,t)\cdot v(r_j,t)}{v(r_j,t)\cdot v(r_j,t)} \tag{S18}$$

where T is the number of frames evaluated. Here the average inside the sum is over space as defined by the variable r'. This correlation was evaluated in Fourier space to reduce computation time. We measured a correlation length scale λ, defined as the length scale at which $A_{vel}(r)$ has decayed to half of its initial amplitude. In contrast to studies of truncated kinesin-1, increasing kinesin-4 concentration increased the velocity-velocity correlation length scale λ [Fig. S10(d)] [41].

I. Modifications of contraction phenomena due to boundary conditions

The dynamics and final structure of a global contraction are sensitive to microtubule concentration, kinesin concentration, initial microtubule alignment, and boundary conditions. When the material is pinned at the ends of the chamber the resulting global contraction displays significant phenomenological differences from its unpinned form [Fig. S13(a)]. First contracting material pinned at the ends of the chamber contract to a thin line. Then the line of material buckles, and then the line of material straightens again. Finally, at long time scales, material accumulates at intervals along the line of the contraction, forming large aster-like clumps. Similarly, non-specific adhering to the chamber sides changed the form of the contraction [Fig. S13(b), (c)].

J. Limits of the kinesin concentration

"Outside the range of experiments shown, we were unable to consistently generate and study active systems. In samples between 0-50 nM kinesin-4 the microtubule tended to remain homogeneous throughout the sample. Samples above 300 nM kinesin-4 lost activity very, within a few minutes. Samples above 2% PEG showed little to no kinesin-driven activity and resulted in slow bulk contractions with or without kinesin."

II. SUPPLEMENTARY VIDEOS

- Video 1: At low microtubule concentrations, dynamic asters spontaneously assemble. This video shows orthogonal planes projected over 6.5 μm. Sample is created with 200 nM kinesin-4 (blue), 400 nM tubulin (black).
- Video 2: At intermediate microtubule density, networks of microtubules globally contract. This video shows four fields of epifluorescent imaging of fluorescent microtubules stitched together. The sample is created with 50 nM kinesin-4 (blue), 1000 nM tubulin (black).
- Video 3: Sedimented asters do not merge and globally contract. This video shows a 3D projection from confocal stacks, with two xy slices at indicated positions. These slices are z-projected over 6.5 μm. Sample is created in a 300 μm chamber with 200 nM kinesin-4 (blue), 400 nM tubulin (black).
- Video 4: At high microtubule density, global contractions align and then roughen. This video shows a z-projection from confocal data, along with xz and yz orthogonal slices projected over 6.5 μm. Starting at 110 min, a 3D surface (blue) generated from the dense surface of the condensate is displayed. This surface shows the location of the orthogonal slices. Sample consisted of 10 μM tubulin (black) and 200 nM kinesin (blue).
- Video 5: At the highest microtubule density, kinesin-4 drives microtubule condensation and the subsequent formation of an active foam. This video shows a 3D projection from confocal stacks of a 333x333x100 μm field of view. Intermittent pauses in the video show the interior structure of the material during its development. Sample contained 200 nM kinesin (blue), 40 μM tubulin (black).
- Video 6: Whole-chamber epifluorescent imaging of highest-density microtubule systems buckling, condensing, and forming an active foam. Sample constituted from 200 nM kinesin (blue), 40 μM tubulin (black).
- Video 7: A series of videos shows a titration of microtubule concentrations in the presence of PEG, resulting in a transition from extensile to contracting networks. All videos are epifluorescent imaging of fluorescent microtubules.
- Video 8: Two videos of extensile networks transforming into bilayer structures. The first video is epifluorescent imaging of fluorescent microtubules (black) and kinesin (blue). The second video is a max-z projection of confocal imaging of a sample in a thin (30 μm) chamber.

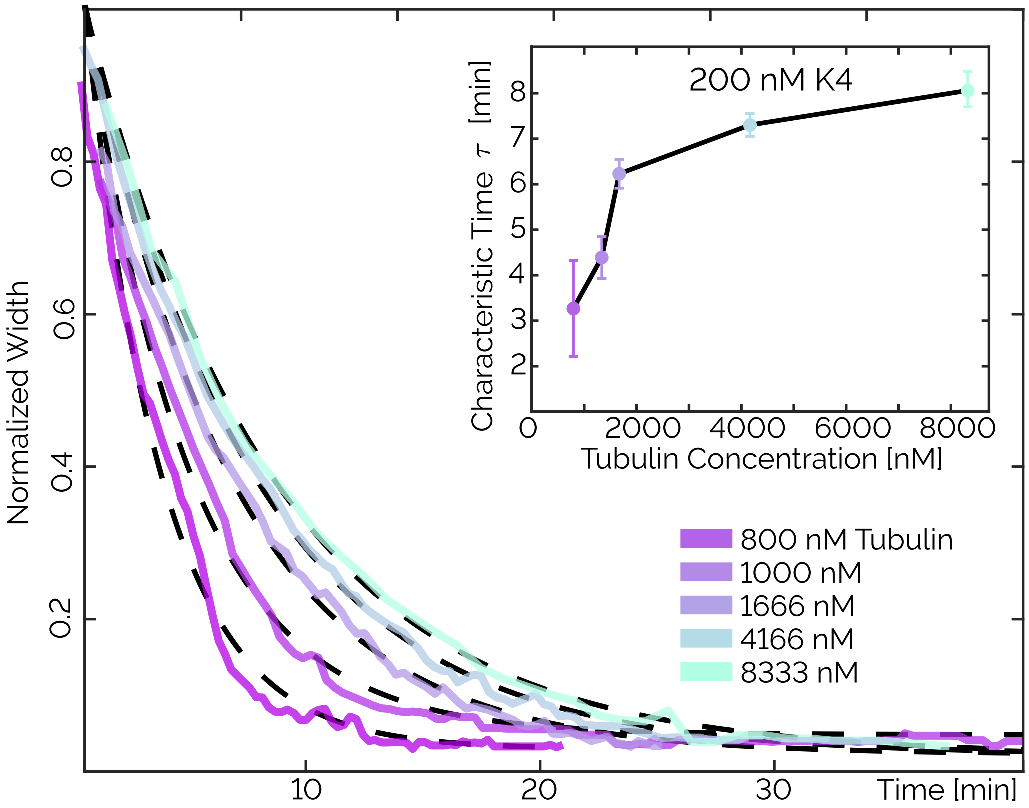

FIG. S1. The contraction time scale decreased with increasing microtubule number density. Plotted is the normalized width W_n at five tubulin concentrations (200 nM kinesin). Dashed lines represent the exponential fit $f_c(t)$. Inset) Characteristic time τ for each tubulin concentration.

III. SUPPLEMENTARY FIGURES

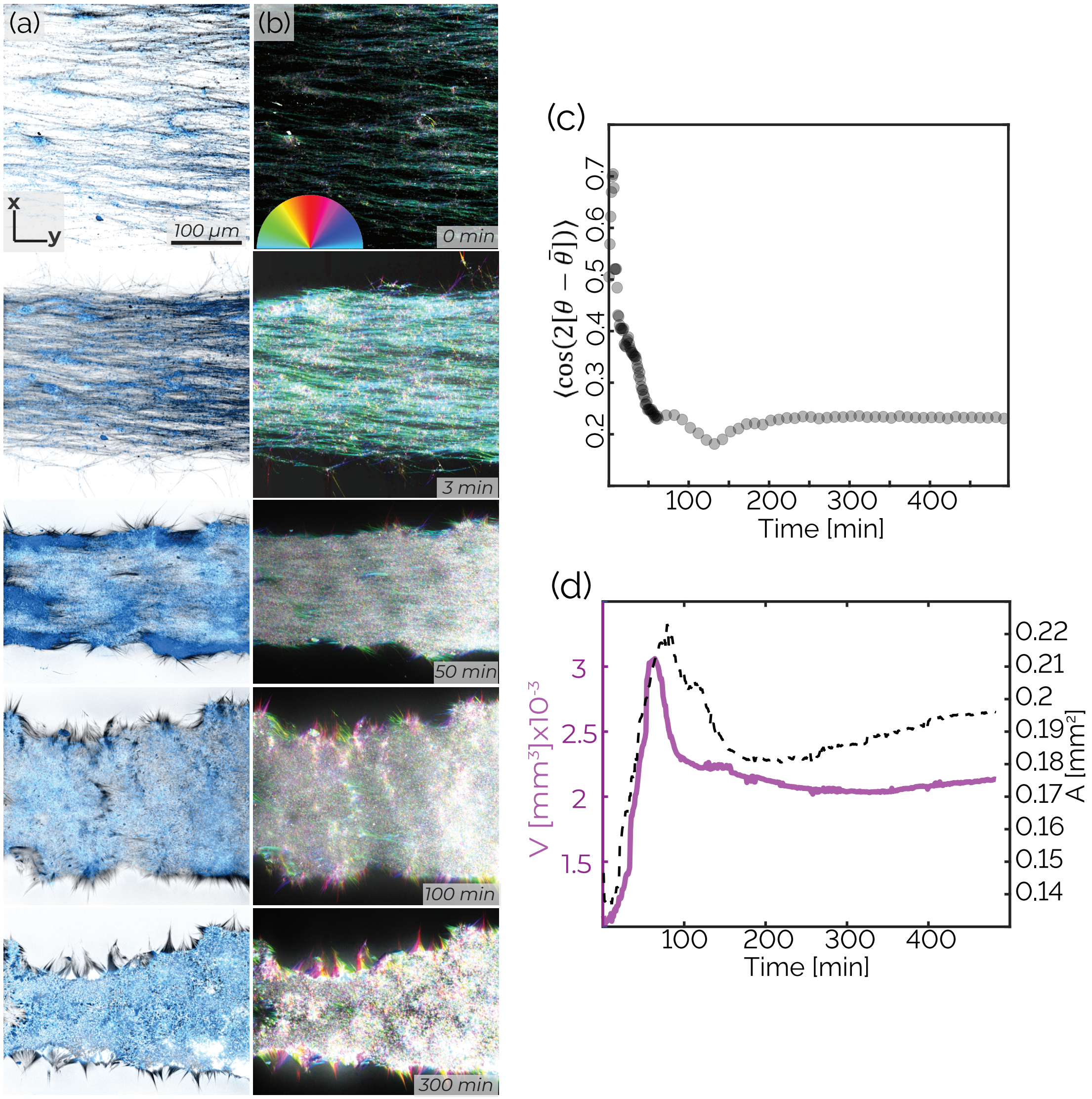

FIG. S2. Replicate 2: Nematic alignment and surface roughening of a contracting network. (a) z-projected images demonstrate that decreasing network volume leads to increasing nematic alignment. (b) z-projection of the microtubule nematic order. Hue indicates the nematic director indicated by the color wheel, while intensity indicates coherency. (c) The microtubule nematic order parameter increases during contraction and then decreases during roughening. (d) The contracting network's volume (solid purple) decreases continuously. Its surface area (dashed black) initially decreases but then increases. Sample composed of 10 μM tubulin (black), 200 nM kinesin (blue).

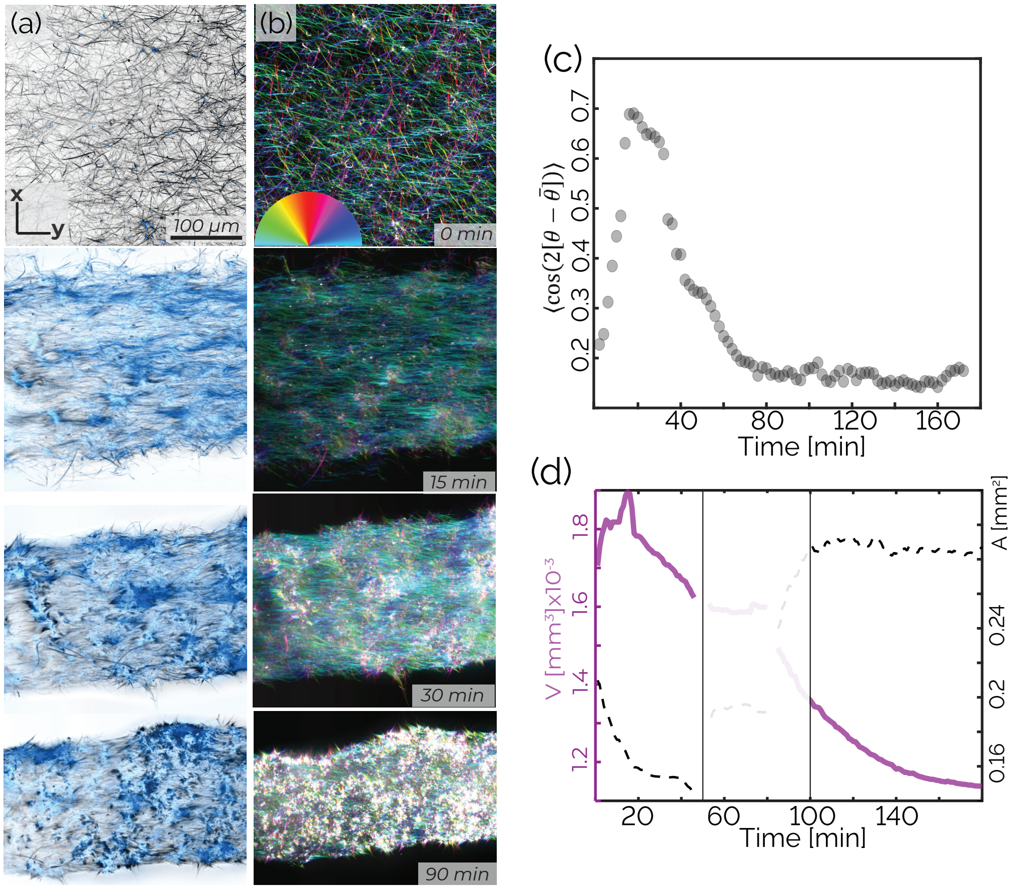

FIG. S3. Replicate 3: Nematic alignment and surface roughening of a contracting network. (a) z-projected images demonstrate that decreasing network volume leads to increasing nematic alignment. (b) z-projection of the microtubule nematic order. Hue indicates the nematic director indicated by the color wheel, while intensity indicates coherency. (c) The microtubule nematic order parameter increases during contraction and then decreases during roughening. (d) The contracting network's volume (solid purple) decreases continuously. Its surface area (dashed black) initially decreases but then increases. Sample composed of 10 μM tubulin (black), 200 nM kinesin (blue).

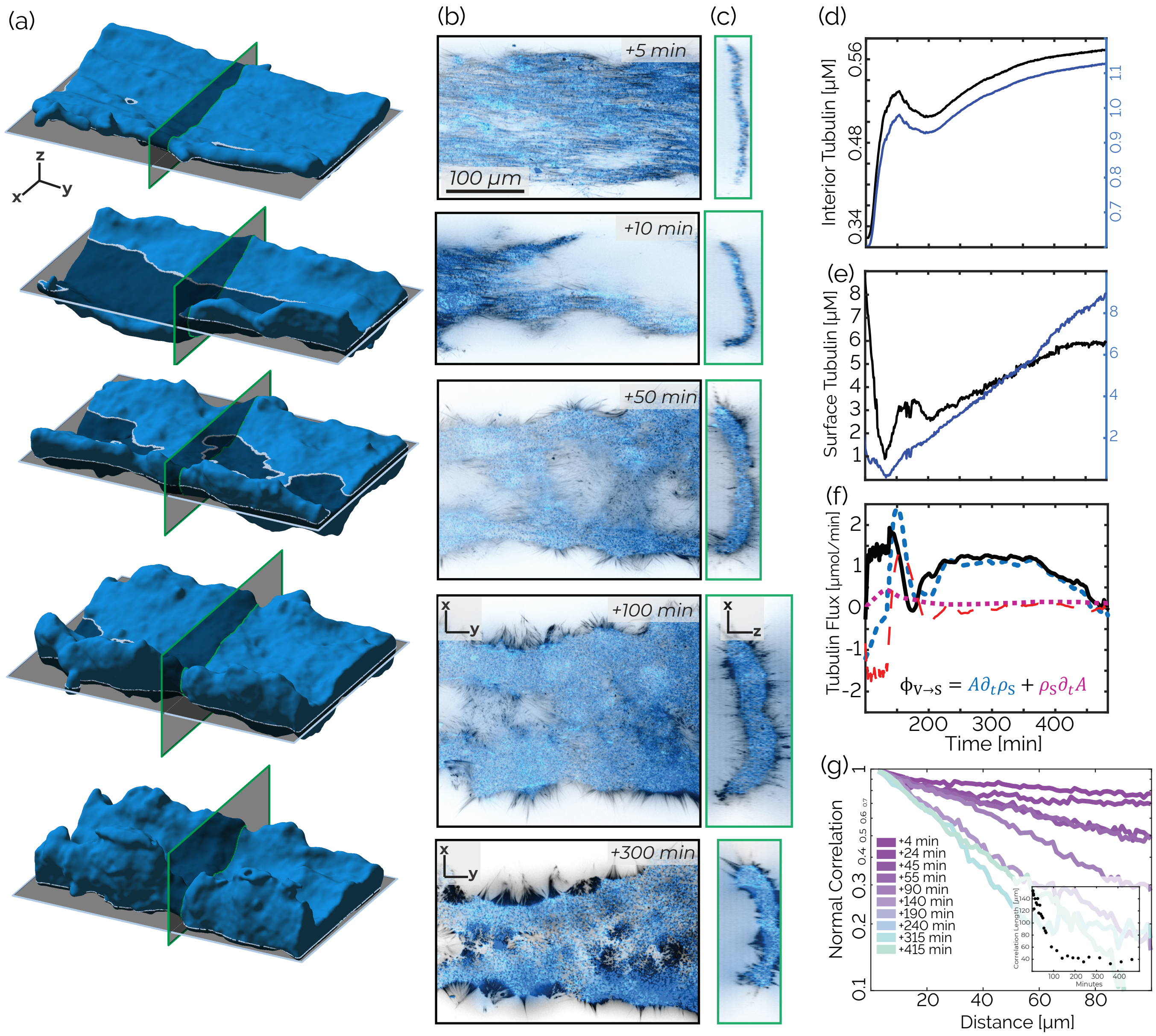

FIG. S4. Replicate 2: A surface roughening accompanied by the formation of a surface-bound monolayer. (a) Time series of a surface of a contracting network. (b) x-y slices of data corresponding to cuts shown in the previous panel. (c) x-z slices show contracting cross-section and highlight the formation of a monolayer. (d) Changes of tubulin and kinesin density within the interior of the contracting network. The sample rapidly contracted in the y-direction at +50 minutes, bringing a different portion of the material into the field of view. This is indicated by a gray overlay in the data. (e) Tubulin and kinesin density within 5 μm of the surface increase during the roughening phase. (f) The flux of microtubules from the interior to the surface $\Phi_{V\to S}$ (black solid), the microtubule surface density $A\partial_t\rho_s$ (blue dashed) and the change in surface area $\rho_s\partial_t A$ (purple short-dashed) as a function of time. The red long-dashed line indicates the sum of all three terms. (g) Normal-normal spatial correlations show faster decay as the material roughens. These correlations are calculated only on a bisected surface, to reduce the influence of the overall surface curvature. Inset: Exponential fits to the normal-normal correlation decay between 10-20 μm. Sample consisted of 10 μM tubulin (black), 200 nM kinesin (blue).

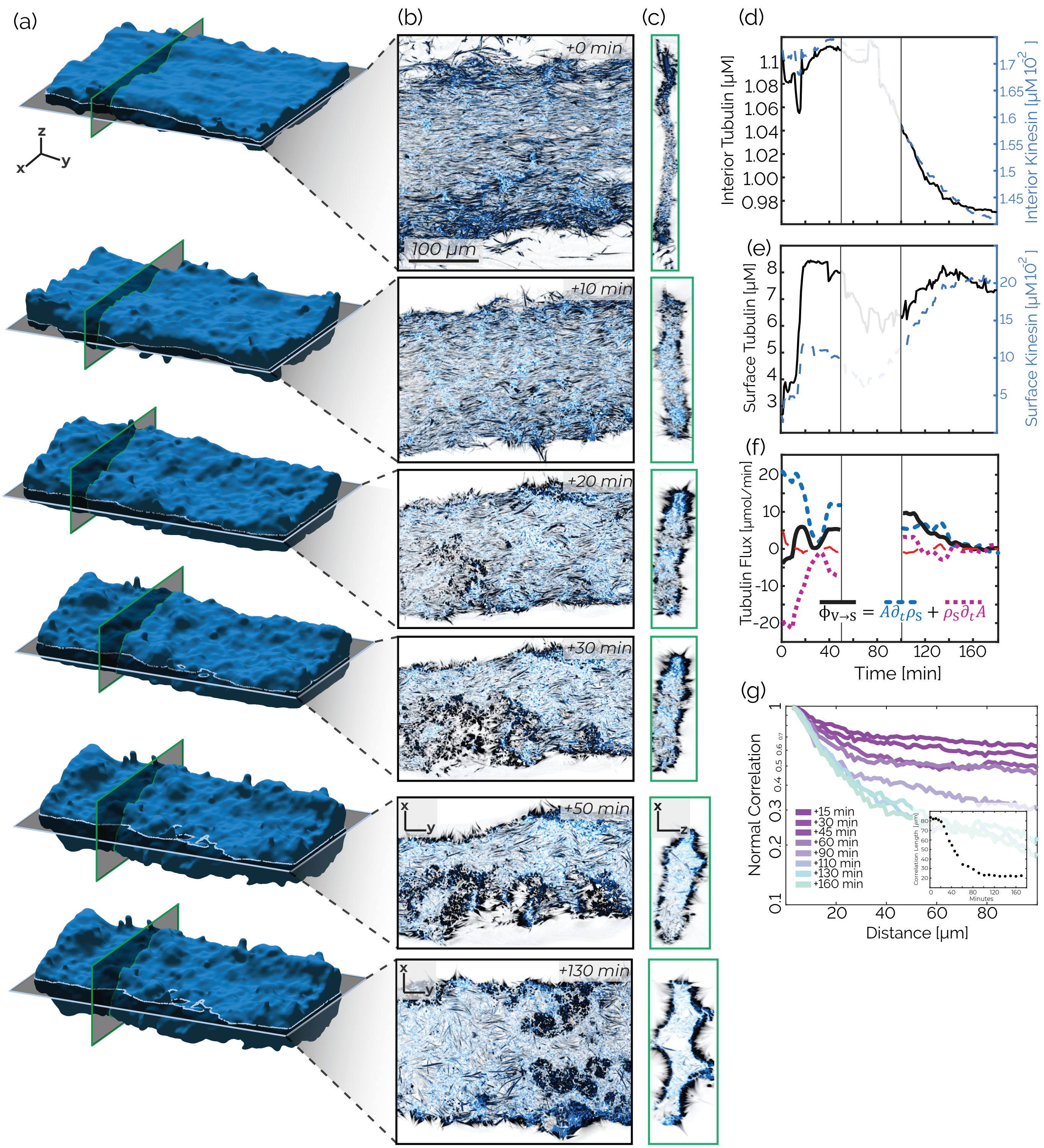

FIG. S5. Replicate 3: A surface roughening accompanied by the formation of a surface-bound monolayer. (a) Time series of a surface of a contracting network. (b) x-y slices of data corresponding to cuts shown in the previous panel. (c) x-z slices show contracting cross-section and highlight the formation of a monolayer. (d) Changes of tubulin and kinesin density within the interior of the contracting network. (e) Tubulin and kinesin density within 5 μm of the surface increase during the roughening phase. (f) The flux of microtubules from the interior to the surface $\Phi_{V\to S}$ (black solid), the microtubule surface density $A\partial_t\rho_s$ (blue dashed) and the change in surface area $\rho_s\partial_t A$ (purple short-dashed) as a function of time. The red long-dashed line indicates the sum of all three terms. Fluxes are not calculated during a period where the sample was rapidly contracting along the y-direction, this section is grayed out and indicated with black horizontal lines. (g) Normal-normal spatial correlations show faster decay as the material roughens. These correlations are calculated only on a bisected surface, to reduce the influence of the overall surface curvature. Inset: Exponential fits to the normal-normal correlation decay between 10-20 μm. Sample consisted of 10 μM tubulin (black), 200 nM kinesin (blue).

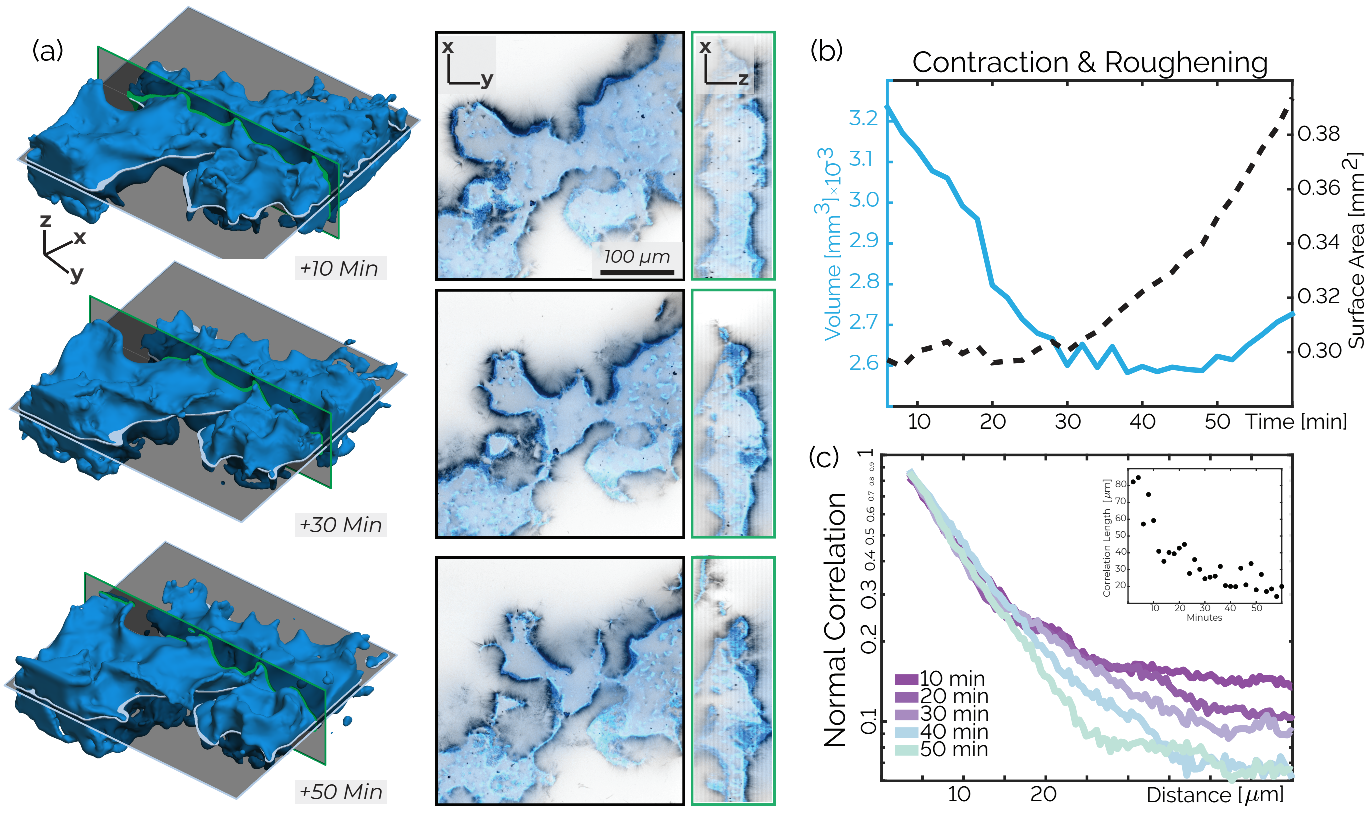

FIG. S6. Replicate 2: Roughening at the highest microtubule concentrations. (a) Evolution of a contracting condensate surface (left) x-y and x-z image cross-sections (right). (b) The volume (solid blue curve) and surface area (black dashed curve) of a contracting condensate as a function of time. (d) The spatial correlation between surface normal vectors decay over time. Inset: Exponential fits to the normal-normal correlation decay between 5-10 μm. Sample contained 200 nM kinesin (blue), 40 μM tubulin (black).

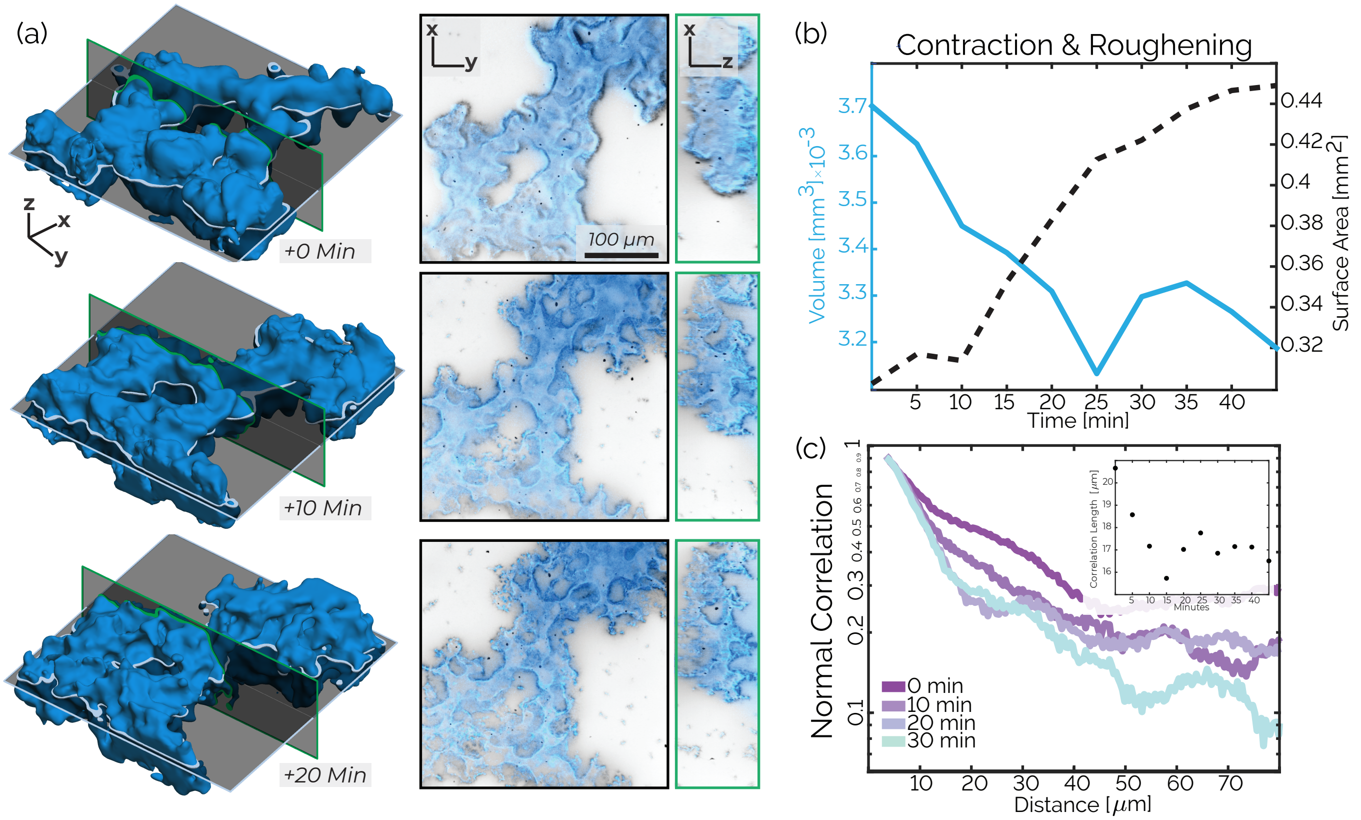

FIG. S7. Replicate 3: Roughening at the highest microtubule concentrations. (a) Evolution of a contracting condensate surface (left) x-y and x-z image cross-sections (right). (b) The volume (solid blue curve) and surface area (black dashed curve) of a contracting condensate as a function of time. (d) The spatial correlation between surface normal vectors decay over time. Inset: Exponential fits to the normal-normal correlation decay between 5-10 μm. Sample contained 200 nM kinesin (blue), 40 μM tubulin (black).

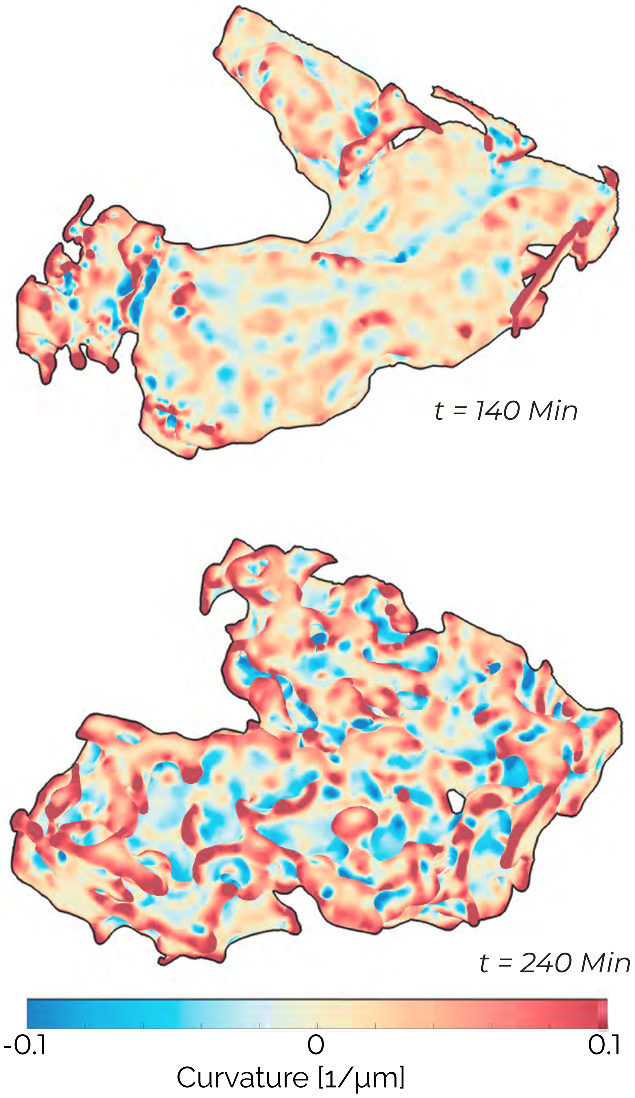

FIG. S8. The mean curvature of the condensate surface shown in Fig. 6 at two points in time, the first after early monolayer formation, the second at the onset of bilayer formation.

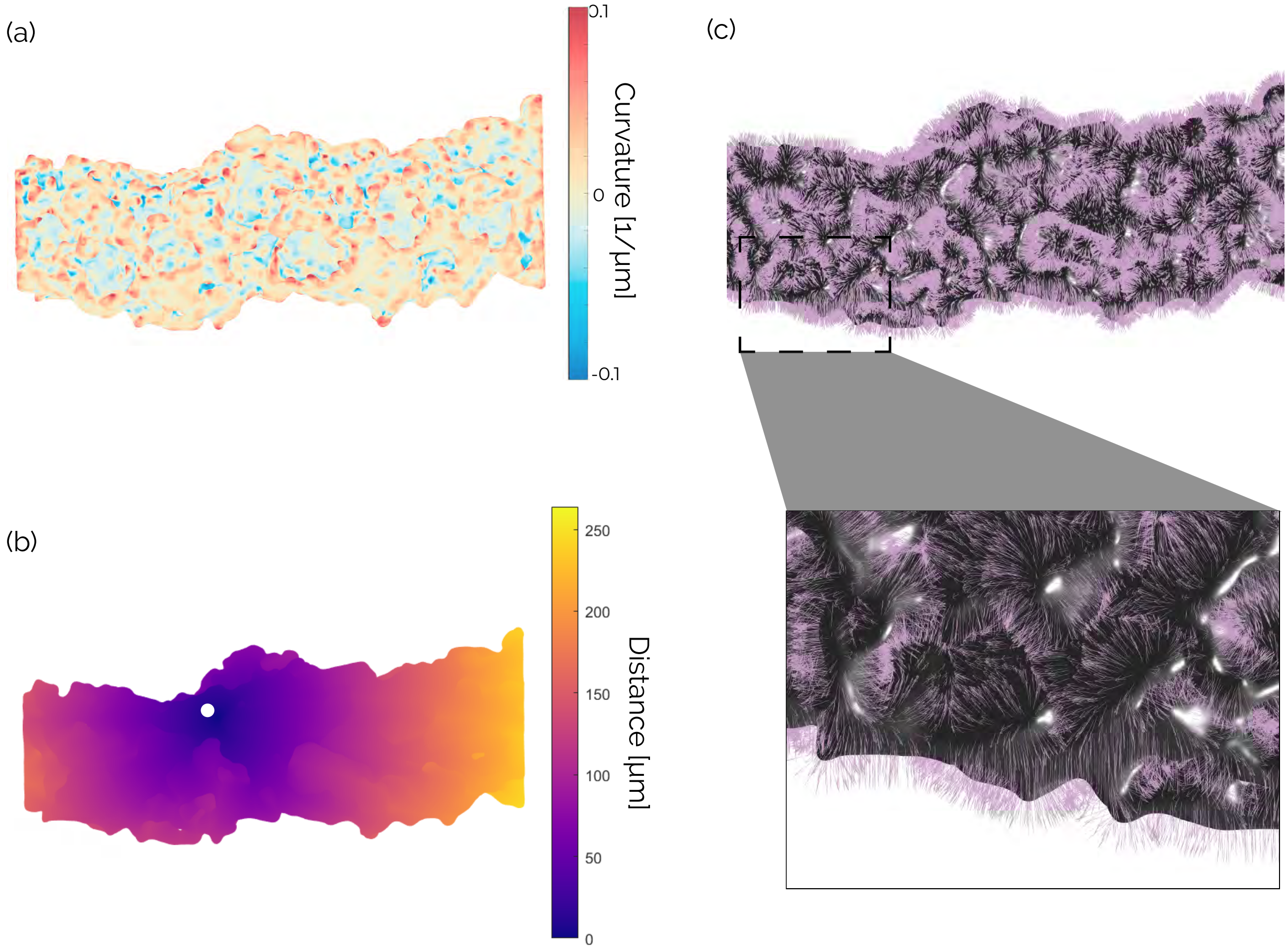

FIG. S9. During roughening, we quantify the normal-normal correlation as a function of geodesic distance along the material surface. (a) Mean curvature of a globally contracting surface at late time. (b) Geodesic distance, on the material surface, from an initial point indicated by a white circle. (c) Lilac arrows indicate normal vectors on the material surface, a random sampling of 10% of the normal vectors are displayed. The ends of the material along the long axis are cropped off for the calculation of normals.

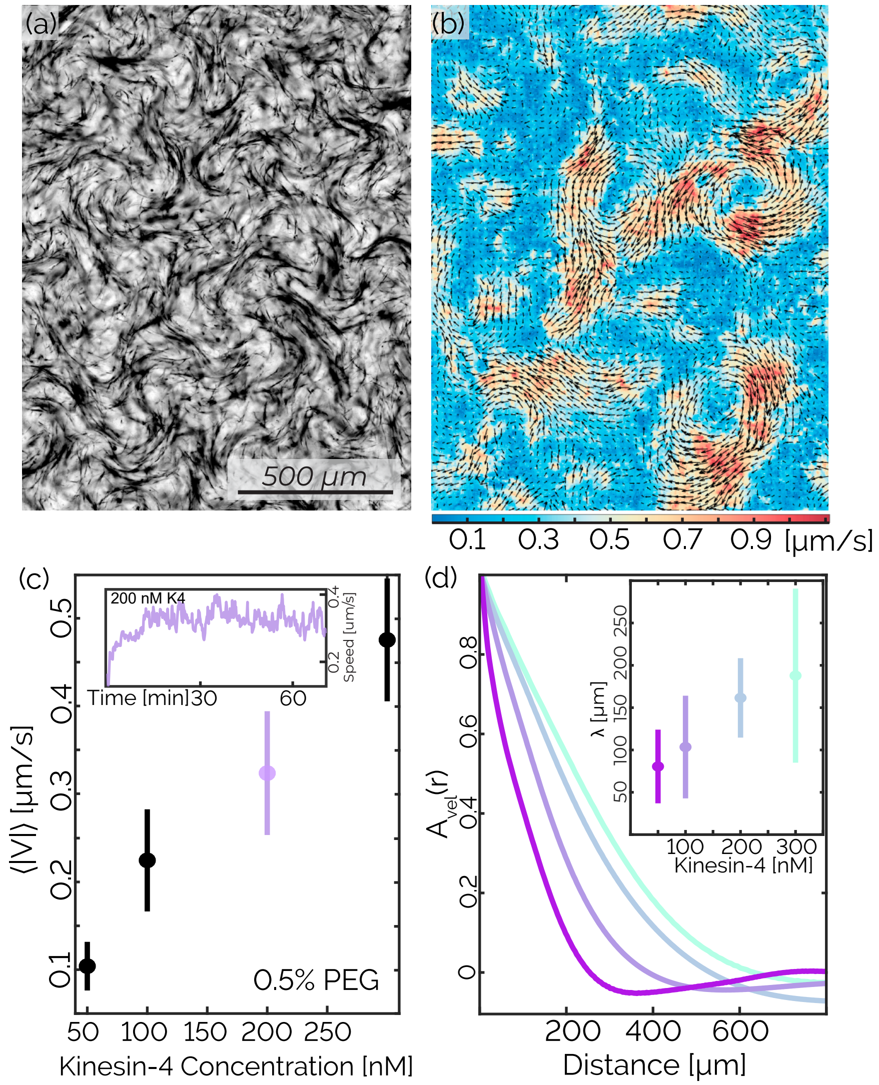

FIG. S10. Increasing kinesin concentration amplifies the dynamics of an extensile fluid. (a) An extensile network driven by kinesin. Sample is created with 200 nM kinesin, 13 μM tubulin (imaged), 0.5% PEG. (b) Color map indicating the magnitude of the material velocity in the previous panel, with overlaid arrows representing the velocity vector field $\vec{v}(r)$. (c) Mean speed $\langle |V| \rangle$ as a function of kinesin. The error bars are standard deviation (n=3). Inset) Spatially averaged speed $U(t) = \langle \vec{v}(r,t) \rangle$ plotted over time for the experiment shown in panel (a). (d) Time-averaged velocity autocorrelation $A(r)$ as a function of kinesin. Inset) Length scale λ at which $2A(\lambda) = A(0)$. Error bars are standard deviation (n=3).

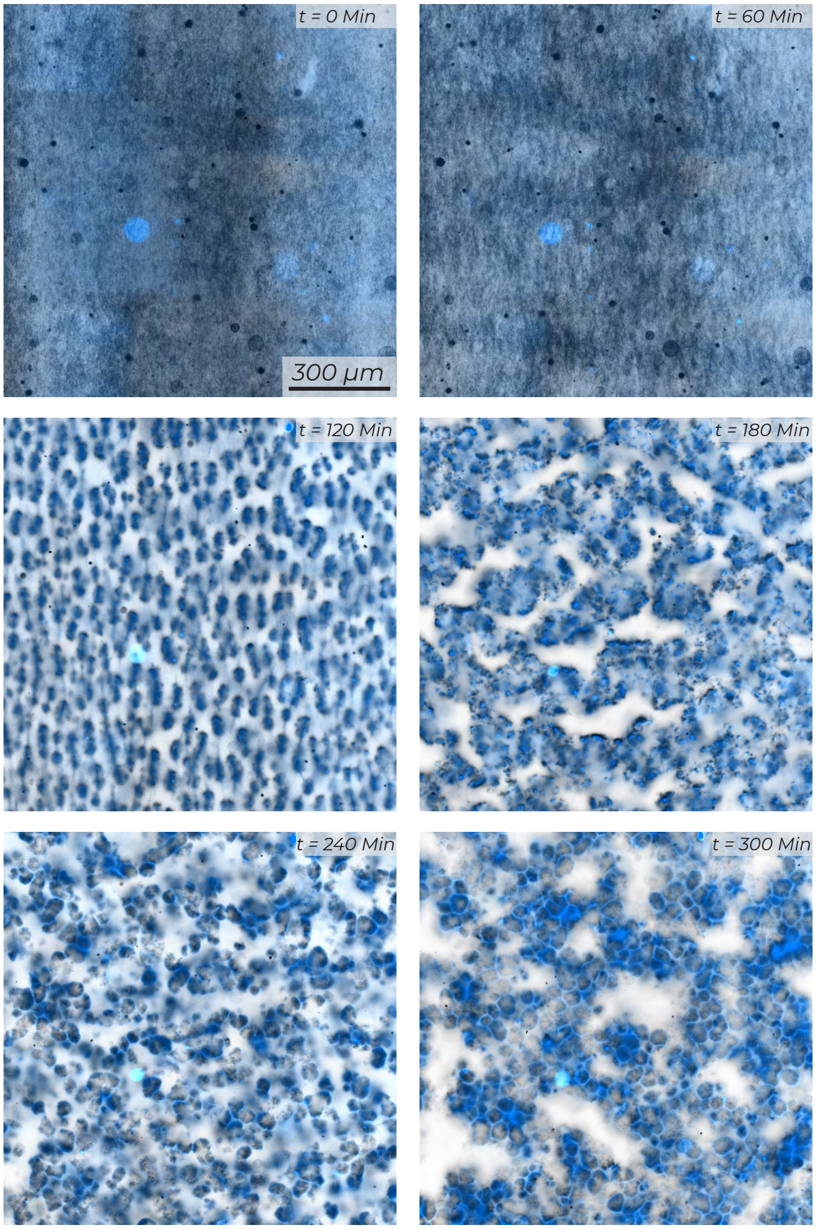

FIG. S11. Low magnification imaging shows the slight buckling and splay of microtubules into monolayer envelopes, followed by the deformation of monolayers into an active bilayer foam.

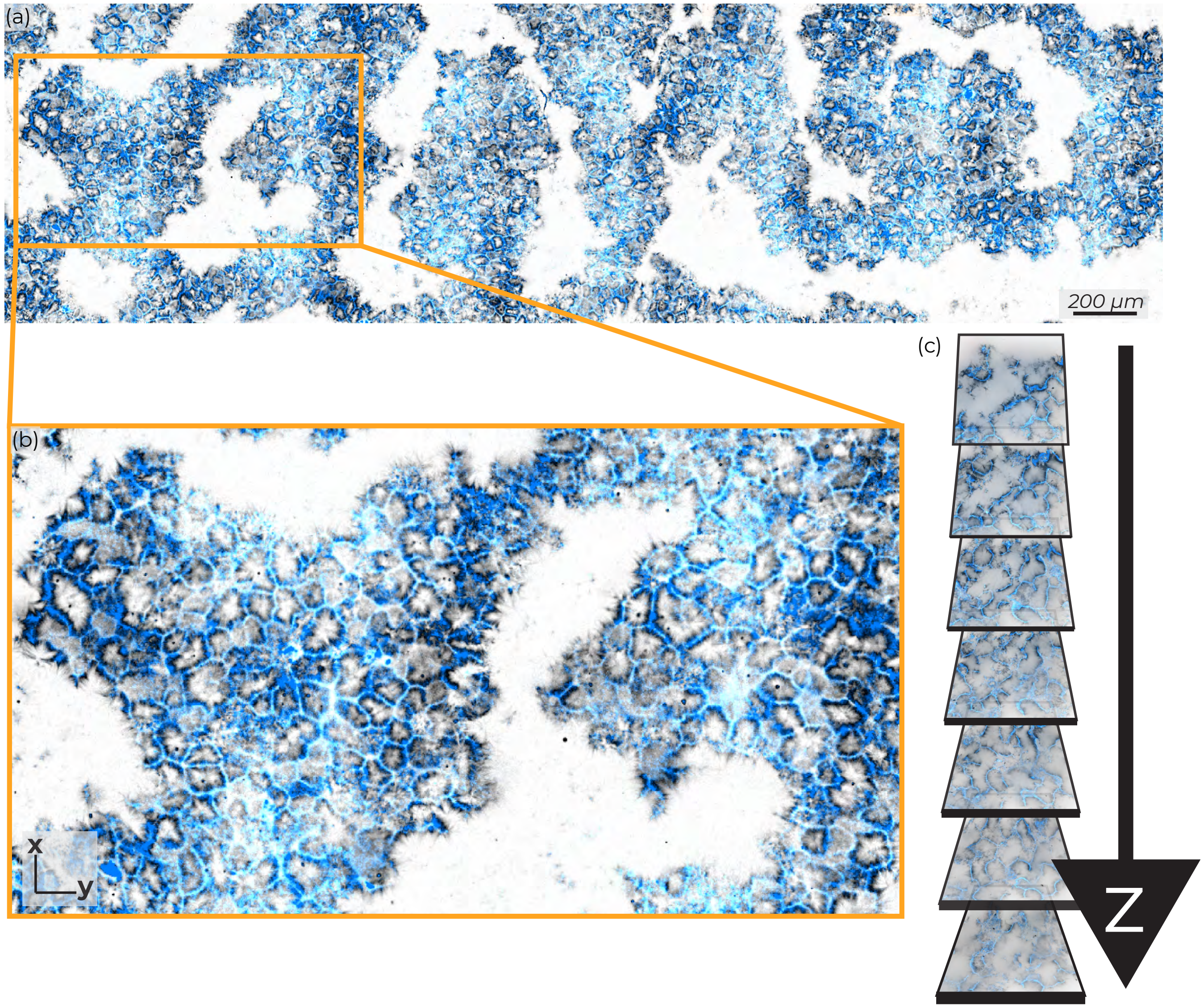

FIG. S12. At high MT concentrations, the mixture coarsens into an active foam. (a) Maximum intensity projection over 10 μm in z of an entire chamber of foam. (b) zoom in (c) Z-stack of 6.5 μm z-projection slices with an additional 6.5 μm in between each slice, showing the 3D structure of a bilayer foam.

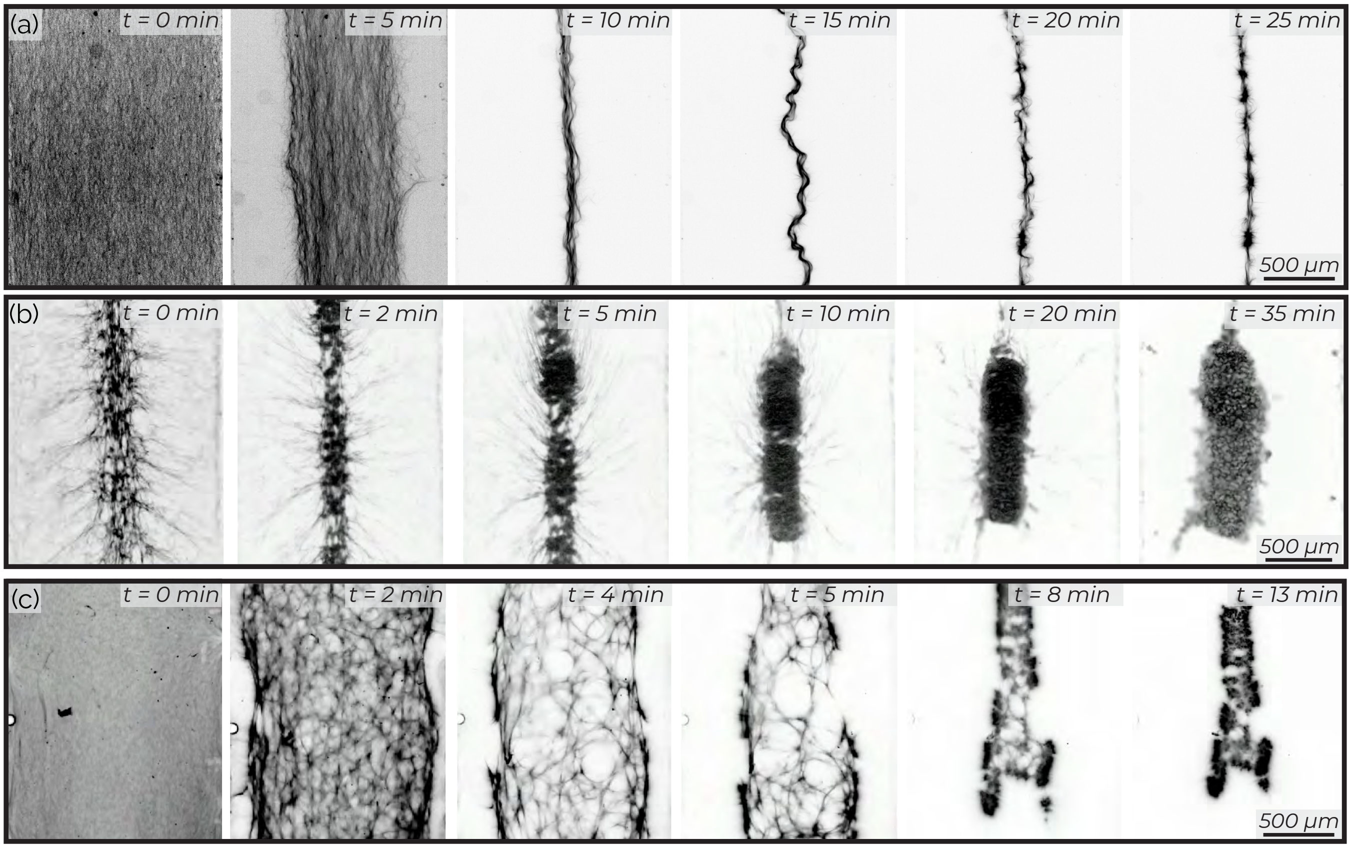

FIG. S13. The behavior of a globally contracting system is influenced by the conditions at the borders of the chamber. (a) A contracting material pinned at the ends of the chamber many millimeters away. This material first contracts but then buckles at 15 min, followed by straightening again at 20 min. (b) A global contraction with some sticking at the parafilm chamber edges. This sample forms an active bilayer foam as its end state. (c) A global contraction loses the symmetry imparted on it by the chamber.

[1] M. C. Marchetti, J. F. Joanny, S. Ramaswamy, T. B. Liverpool, J. Prost, M. Rao, and R. A. Simha, Hydrodynamics of soft active matter, Reviews of Modern Physics **85**, 1143 (2013).
[2] F. Nedelec, T. Surrey, A. C. Maggs, and S. Leibler, Self-organization of microtubules and motors, Nature **389**, 305 (1997).
[3] V. Schaller, C. Weber, C. Semmrich, E. Frey, and A. R. Bausch, Polar patterns of driven filaments, Nature **467**, 73 (2010).
[4] A. Bricard, J.-B. Caussin, N. Desreumaux, O. Dauchot, and D. Bartolo, Emergence of macroscopic directed motion in populations of motile colloids, Nature **503**, 95 (2013).
[5] V. Narayan, S. Ramaswamy, and N. Menon, Long-lived giant number fluctuations in a swarming granular nematic, Science **317**, 105 (2007).
[6] V. Soni, E. S. Bililign, S. Magkiriadou, S. Sacanna, D. Bartolo, M. J. Shelley, and W. T. Irvine, The odd free surface flows of a colloidal chiral fluid, Nature Physics **15**, 1188 (2019).
[7] I. Theurkauff, C. Cottin-Bizonne, J. Palacci, C. Ybert, and L. Bocquet, Dynamic clustering in active colloidal suspensions with chemical signaling, Physical review letters **108**, 268303 (2012).
[8] J. Palacci, S. Sacanna, A. P. Steinberg, D. J. Pine, and P. M. Chaikin, Living crystals of light-activated colloidal surfers, Science **339**, 936 (2013).
[9] G. S. Redner, M. F. Hagan, and A. Baskaran, Structure and dynamics of a phase-separating active colloidal fluid, Physical review letters **110**, 055701 (2013).
[10] Y. Fily and M. C. Marchetti, Athermal phase separation of self-propelled particles with no alignment, Physical review letters **108**, 235702 (2012).
[11] C. Thomas, T. Surrey, F. Nédélec, J. Rickman, and J. Roostalu, Determinants of polar versus nematic organization in networks of dynamic microtubules and mitotic motors, Cell **175**, 796 (2018).
[12] C. Dombrowski, L. Cisneros, S. Chatkaew, R. E. Goldstein, and J. O. Kessler, Self-concentration and large-scale coherence in bacterial dynamics, Physical review letters **93**, 098103 (2004).
[13] T. Sanchez, D. T. N. Chen, S. J. DeCamp, M. Heymann, and Z. Dogic, Spontaneous motion in hierarchically assembled active matter, Nature **491**, 431 (2012).
[14] S. Zhou, A. Sokolov, O. D. Lavrentovich, and I. S. Aranson, Living liquid crystals, Proceedings of the National Academy of Sciences **111**, 1265 (2014).
[15] P. M. Bendix, G. H. Koenderink, D. Cuvelier, Z. Dogic, B. N. Koeleman, W. M. Brieher, C. M. Field, L. Mahadevan, and D. A. Weitz, A quantitative analysis of contractility in active cytoskeletal protein networks, Biophysical journal **94**, 3126 (2008).
[16] P. J. Foster, S. Furthauer, M. J. Shelley, and D. J. Needleman, Active contraction of microtubule networks, eLife **4**, 1 (2015).
[17] T. B. Liverpool and M. C. Marchetti, Bridging the microscopic and the hydrodynamic in active filament solutions, EPL (Europhysics Letters) **69**, 846 (2005).
[18] T. Gao, R. Blackwell, M. A. Glaser, M. D. Betterton, and M. J. Shelley, Multiscale polar theory of microtubule and motor-protein assemblies, Physical review letters **114**, 048101 (2015).
[19] G. A. Vliegenthart, A. Ravichandran, M. Ripoll, T. Auth, and G. Gompper, Filamentous active matter: Band formation, bending, buckling, and defects, Science advances **6**, 9975 (2020).
[20] J. M. Belmonte, M. Leptin, and F. Nédélec, A theory that predicts behaviors of disordered cytoskeletal networks, Molecular Systems Biology **13**, 941 (2017).
[21] M. Lenz, Reversal of contractility as a signature of self-organization in cytoskeletal bundles, Elife **9**, 51751 (2020).
[22] D. Needleman and Z. Dogic, Active matter at the interface between materials science and cell biology, Nature Reviews Materials **2**, 10.1038/natrevmats.2017.48 (2017).
[23] J. N. Israelachvili, D. J. Mitchell, and B. W. Ninham, Theory of self-assembly of hydrocarbon amphiphiles into micelles and bilayers, Journal of the Chemical Society, Faraday Transactions 2: Molecular and Chemical Physics **72**, 1525 (1976).
[24] F. S. Bates and G. H. Fredrickson, Block copolymer thermodynamics: theory and experiment, Annual review of physical chemistry **41**, 525 (1990).
[25] S. Safran, *Statistical thermodynamics of surfaces, interfaces, and membranes* (CRC Press, 2018).
[26] P. Bieling, I. A. Telley, and T. Surrey, A minimal midzone protein module controls formation and length of antiparallel microtubule overlaps, Cell **142**, 420 (2010).
[27] R. Subramanian, S. C. Ti, L. Tan, S. A. Darst, and T. M. Kapoor, Marking and measuring single microtubules by prc1 and kinesin-4, Cell **154**, 377 (2013).
[28] S. Wijeratne and R. Subramanian, Geometry of antiparallel microtubule bundles regulates relative sliding and stalling by prc1 and kif4a, Elife **7**, 10.7554/eLife.32595 (2018).
[29] C.-K. Hu, M. Coughlin, C. M. Field, and T. J. Mitchison, Kif4 regulates midzone length during cytokinesis, Current Biology **21**, 815 (2011).
[30] T. J. Mitchison, P. Nguyen, M. Coughlin, and A. C. Groen, Self-organization of stabilized microtubules by both spindle and midzone mechanisms in xenopus egg cytosol, Molecular Biology of the Cell **24**, 1559 (2013).
[31] S. Wijeratne, S. A. Fiorenza, R. Subramanian, and M. Betterton, Motor guidance by long-range communication through the microtubule highway, bioRxiv (2020).
[32] R. Tan, P. J. Foster, D. J. Needleman, and R. J. McKenney, Cooperative accumulation of dynein-dynactin at microtubule minus-ends drives microtubule network reorganization, Developmental Cell **44**, 233 (2018).

[33] P. Marquez-Neila, L. Baumela, and L. Alvarez, A morphological approach to curvature-based evolution of curves and surfaces, IEEE Transactions on Pattern Analysis and Machine Intelligence **36**, 2 (2014).
[34] T. F. Chan and L. A. Vese, Active contours without edges, IEEE Transactions on image processing **10**, 266 (2001).
[35] S. Osher and R. Fedkiw, *Level set methods and dynamic implicit surfaces*, Vol. 153 (Springer Science & Business Media, 2006).
[36] R. A. Simha and S. Ramaswamy, Hydrodynamic fluctuations and instabilities in ordered suspensions of self-propelled particles, Physical review letters **89**, 058101 (2002).
[37] P. Chandrakar, J. Berezney, B. Lemma, B. Hishamunda, A. Berry, K. T. Wu, R. Subramanian, J. Chung, D. Needleman, J. Gelles, and Z. Dogic, Microtubule-based active fluids with improved lifetime, temporal stability and miscibility with passive soft materials, arXiv (2018).
[38] A. Ward, F. Hilitski, W. Schwenger, D. Welch, A. W. Lau, V. Vitelli, L. Mahadevan, and Z. Dogic, Solid friction between soft filaments, Nat Mater **14**, 583 (2015).
[39] S. Ramaswamy, The mechanics and statistics of active matter, Annu. Rev. Condens. Matter Phys. **1**, 323 (2010).
[40] P. Chandrakar, M. Varghese, S. A. Aghvami, A. Baskaran, Z. Dogic, and G. Duclos, Confinement controls the bend instability of three-dimensional active liquid crystals, Physical Review Letters **125**, 257801 (2020).
[41] G. Henkin, S. J. DeCamp, D. T. N. Chen, T. Sanchez, and Z. Dogic, Tunable dynamics of microtubule-based active isotropic gels, Philosophical Transactions of the Royal Society A: Mathematical, Physical and Engineering Sciences **372**, 10.1098/rsta.2014.0142 (2014).
[42] M. P. Murrell and M. L. Gardel, F-actin buckling coordinates contractility and severing in a biomimetic actomyosin cortex, Proceedings of the National Academy of Sciences **109**, 20820 (2012).
[43] M. S. e Silva, M. Depken, B. Stuhrmann, M. Korsten, F. C. MacKintosh, and G. H. Koenderink, Active multistage coarsening of actin networks driven by myosin motors, Proceedings of the National Academy of Sciences **108**, 9408 (2011).
[44] S. Stam, S. L. Freedman, S. Banerjee, K. L. Weirich, A. R. Dinner, and M. L. Gardel, Filament rigidity and connectivity tune the deformation modes of active biopolymer networks, Proceedings of the National Academy of Sciences **114**, 10037 (2017).
[45] N. Kumar, R. Zhang, J. J. De Pablo, and M. L. Gardel, Tunable structure and dynamics of active liquid crystals, Science advances **4**, 7779 (2018).
[46] J. Berezney, B. L. Goode, S. Fraden, and Z. Dogic, Extensile to contractile transition in active microtubule–actin composites generates layered asters with programmable lifetimes, Proceedings of the National Academy of Sciences **119**, e2115895119 (2022).
[47] S. Fürthauer and M. J. Shelley, How cross-link numbers shape the large-scale physics of cytoskeletal materials, Annual Review of Condensed Matter Physics **13**, 365 (2022).
[48] S. Fürthauer, B. Lemma, P. J. Foster, S. C. Ems-McClung, C.-H. Yu, C. E. Walczak, Z. Dogic, D. J. Needleman, and M. J. Shelley, Self-straining of actively crosslinked microtubule networks, Nature physics **15**, 1295 (2019).
[49] L. M. Lemma, M. M. Norton, A. M. Tayar, S. J. DeCamp, S. A. Aghvami, S. Fraden, M. F. Hagan, and Z. Dogic, Multiscale microtubule dynamics in active nematics, Physical Review Letters **127**, 148001 (2021).
[50] R. Blackwell, O. Sweezy-Schindler, C. Baldwin, L. E. Hough, M. A. Glaser, and M. Betterton, Microscopic origins of anisotropic active stress in motor-driven nematic liquid crystals, Soft Matter **12**, 2676 (2016).
[51] P. Ronceray, C. P. Broedersz, and M. Lenz, Fiber networks amplify active stress, Proceedings of the national academy of sciences **113**, 2827 (2016).
[52] C. Hentrich and T. Surrey, Microtubule organization by the antagonistic mitotic motors kinesin-5 and kinesin-14, Journal of Cell Biology **189**, 465 (2010).
[53] T. Surrey, F. Nédélec, S. Leibler, and E. Karsenti, Physical properties determining self-organization of motors and microtubules, Science **292**, 1167 (2001).
[54] K. Kruse, J.-F. Joanny, F. Jülicher, J. Prost, and K. Sekimoto, Asters, vortices, and rotating spirals in active gels of polar filaments, Physical review letters **92**, 78101 (2004).
[55] K. Husain and M. Rao, Emergent structures in an active polar fluid: Dynamics of shape, scattering, and merger, Physical review letters **118**, 078104 (2017).
[56] I. Maryshev, D. Marenduzzo, A. B. Goryachev, and A. Morozov, Kinetic theory of pattern formation in mixtures of microtubules and molecular motors, Physical Review E **97**, 022412 (2018).
[57] I. Maryshev, A. Morozov, A. B. Goryachev, and D. Marenduzzo, Pattern formation in active model c with anchoring: bands, aster networks, and foams, Soft Matter **16**, 8775 (2020).
[58] E. Hannak and R. Heald, Investigating mitotic spindle assembly and function in vitro using xenopus laevis egg extracts, Nature protocols **1**, 2305 (2006).
[59] A. Thawani, H. A. Stone, J. W. Shaevitz, and S. Petry, Spatiotemporal organization of branched microtubule networks, Elife **8**, 43890 (2019).
[60] J. F. Pelletier, C. M. Field, S. Fürthauer, M. Sonnett, and T. J. Mitchison, Co-movement of astral microtubules, organelles and f-actin by dynein and actomyosin forces in frog egg cytoplasm, Elife **9**, e60047 (2020).
[61] F. Verde, J.-M. Berrez, C. Antony, and E. Karsenti, Taxol-induced microtubule asters in mitotic extracts of xenopus eggs: requirement for phosphorylated factors and cytoplasmic dynein, The Journal of cell biology **112**, 1177 (1991).
[62] J. M. Scholey, G. Civelekoglu-Scholey, and I. Brust-Mascher, Anaphase b, Biology **5**, 51 (2016).
[63] M. I. Anjur-Dietrich, C. P. Kelleher, and D. J. Needleman, Mechanical mechanisms of chromosome segregation, Cells **10**, 465 (2021).
[64] C. H. Yu, S. Redemann, H. Y. Wu, R. Kiewisz, T. Y. Yoo, W. Conway, R. Farhadifar, T. Müller-Reichert, and D. Needle-

man, Central-spindle microtubules are strongly coupled to chromosomes during both anaphase a and anaphase b, Molecular Biology of the Cell **30**, 2503 (2019).
[65] J. Hannabuss, M. Lera-Ramirez, N. I. Cade, F. J. Fourniol, F. Nédélec, and T. Surrey, Self-organization of minimal anaphase spindle midzone bundles, Current Biology **29**, 2120 (2019).
[66] B. Martínez-Prat, J. Ignés-Mullol, J. Casademunt, and F. Sagués, Selection mechanism at the onset of active turbulence, Nature physics **15**, 362 (2019).
[67] R. B. Meyer, Macroscopic phenomena in polymer liquid crystals, Molecular Crystals and Liquid Crystals **106**, 414 (1984).
[68] M. Castoldi and A. V. Popov, Purification of brain tubulin through two cycles of polymerization–depolymerization in a high-molarity buffer, Protein expression and purification **32**, 83 (2003).
[69] A. Hyman, D. Drechsel, D. Kellogg, S. Salser, K. Sawin, P. Steffen, L. Wordeman, and T. Mitchison, [39] preparation of modified tubulins, Methods in enzymology **196**, 478 (1991).
[70] J. Howard and R. Clark, Mechanics of motor proteins and the cytoskeleton, Appl. Mech. Rev. **55**, B39 (2002).
[71] A. Lau, A. Prasad, and Z. Dogic, Condensation of isolated semi-flexible filaments driven by depletion interactions, EPL (Europhysics Letters) **87**, 48006 (2009).
[72] J. Bigun, T. Bigun, and K. Nilsson, Recognition by symmetry derivatives and the generalized structure tensor, IEEE Trans Pattern Anal Mach Intell **26**, 1590 (2004).
[73] R. Rezakhaniha, A. Agianniotis, J. T. C. Schrauwen, A. Griffa, D. Sage, C. v. Bouten, F. Van De Vosse, M. Unser, and N. Stergiopulos, Experimental investigation of collagen waviness and orientation in the arterial adventitia using confocal laser scanning microscopy, Biomechanics and modeling in mechanobiology **11**, 461 (2012).
[74] S. Berg, D. Kutra, T. Kroeger, C. N. Straehle, B. X. Kausler, C. Haubold, M. Schiegg, J. Ales, T. Beier, and M. Rudy, Ilastik: interactive machine learning for (bio) image analysis, Nature Methods , 1 (2019).
[75] P. Cignoni, M. Callieri, M. Corsini, M. Dellepiane, F. Ganovelli, and G. Ranzuglia, Meshlab: an open-source mesh processing tool., in *Eurographics Italian chapter conference*, Vol. 2008 (Salerno, Italy, 2008) pp. 129–136.
[76] W. Thielicke and E. Stamhuis, Pivlab–towards user-friendly, affordable and accurate digital particle image velocimetry in matlab, Journal of open research software **2** (2014).
